# Impact of Prescription Medicines on Work-Related Outcomes in Workers with Musculoskeletal Disorders or Injuries: A Systematic Scoping Review

**DOI:** 10.1007/s10926-023-10138-y

**Published:** 2023-11-07

**Authors:** Yonas Getaye Tefera, Shannon Gray, Suzanne Nielsen, Asmare Gelaw, Alex Collie

**Affiliations:** 1 Healthy Working Lives Research Group, School of Public Health and Preventive Medicine, Monash University, 553 St Kilda Road, Melbourne, VIC 3004 Australia; 2 Monash Addiction Research Centre, Eastern Health Clinical School, Monash University, 47-49 Moorooduc Hwy, Frankston, 3199 Australia

**Keywords:** Prescription medicine, Work outcomes, Musculoskeletal disorders, Injuries

## Abstract

**Purpose:**

Medicines are often prescribed to workers with musculoskeletal disorders (MSDs) and injuries to relieve pain and facilitate their recovery and return to work. However, there is a growing concern that prescription medicines may have adverse effects on work function. This scoping review aimed to summarize the existing empirical evidence on prescription medicine use by workers with MSD or injury and its relationship with work-related outcomes.

**Methods:**

We identified studies through structured searching of MEDLINE, EMBASE, PsycINFO, CINAHL Plus, Scopus, Web of Science and Cochrane library databases, and via searching of dissertations, theses, and grey literature databases. Studies that examined the association between prescription medicine and work-related outcomes in working age people with injury or MSDs, and were published in English after the year 2000 were eligible.

**Results:**

From the 4884 records identified, 65 studies were included for review. Back disorders and opioids were the most commonly studied musculoskeletal conditions and prescription medicines, respectively. Most studies showed a negative relationship between prescription medicines and work outcomes. Opioids, psychotropics and their combination were the most common medicines associated with adverse work outcomes. Opioid prescriptions with early initiation, long-term use, strong and/or high dose and extended pre- and post-operative use in workers’ compensation setting were consistently associated with adverse work function. We found emerging but inconsistent evidence that skeletal muscle relaxants and non-steroidal anti-inflammatory drugs were associated with unfavorable work outcomes.

**Conclusion:**

Opioids and other prescription medicines might be associated with adverse work outcomes. However, the evidence is conflicting and there were relatively fewer studies on non-opioid medicines. Further studies with more robust design are required to enable more definitive exploration of causal relationships and settle inconsistent evidence.

**Supplementary Information:**

The online version contains supplementary material available at 10.1007/s10926-023-10138-y.

## Introduction

Musculoskeletal disorders (MSDs) and musculoskeletal injuries refer to a range of conditions affecting the muscles, bones, tendons, ligaments, and other tissues that often result from biological changes, degenerative processes, repetitive movements, overexertion, poor ergonomics, physical harm or damage to the body [1, 2]. MSDs and injuries are leading causes of disease burden globally [3, 4]. Injuries and MSDs disproportionately affect the working age population due to injury vulnerability and exacerbations of musculoskeletal conditions by poor ergonomics or exposure to physical and/or psychosocial hazards in the workplace [5, 6]. MSDs and injuries are the leading cause of work disability [7–9]. Globally, it is estimated that more than one billion people aged 15–64 years with MSDs would benefit from better access to healthcare and rehabilitation [3].

Clinical therapeutic guidelines recommend non-pharmacological treatments such as physical and psychological therapies for MSDs (e.g. low back, neck, and upper-limb pain) and minimise the use of medicines including commonly used analgesics [10, 11]. However, medicines are often prescribed to relieve pain and other symptoms. For example, acetaminophen and non-steroidal anti-inflammatory drugs (NSAIDs) are widely recommended for acute pain management. For severe pain that does not respond to first-line analgesics, a short course of opioid therapy may be considered; however, long-term use is strongly discouraged [11–14]. Despite this, opioids and other pharmacotherapies are frequently prescribed for an extended period [15, 16]. Prolonged use of these medicines has been associated with increased health care costs and longer disability duration [17, 18].

Workers’ compensation and sickness absence systems are established to provide income support (such as replacement of lost wages) and payment of medical expenses and rehabilitation services for workers who have become injured or ill during the course of their employment [19, 20]. In nations with statutory workers’ compensation schemes (e.g., Australia, the United States, Canada, Hong Kong, Japan), injury and MSDs claims account for the majority of workers’ compensation claims and scheme costs. For example, in the 2019/20 financial year 88% of claims exceeding 5 days duration of time off work in Australia were for injuries or MSDs [21]. Similarly, MSDs account for 40% of all lost-time workplace injuries in the United States and Canada [22, 23]. Further, a significant amount of the total medical costs associated with workers’ compensation—between 15% and 20%—goes towards the reimbursement of prescription drugs for injured workers [24, 25]. The substantial financial costs of prescription medicines, and their potential to adversely impact work outcomes has raised concerns among leading clinicians and scholars, and their continued extensive use in these schemes has been criticized [26, 27]. Despite these concerns, the impact of prescription medicines on work outcomes such as functional improvement, time off work or duration of disability has not been comprehensively studied, particularly for non-opioid medicines.

Accumulated evidence demonstrates that opioid prescriptions are associated with adverse work outcomes [18, 27–29]. However, evidence is scarce about the effect on work outcomes of other commonly prescribed medicines including psychotropics, skeletal muscle relaxants (SMRs) and NSAIDs in people with injuries and MSDs. Therefore, this scoping review aimed to: (i) explore and summarize the available evidence on the associations of medicine use and work outcomes in working age people with injury and MSDs; and (ii) identify evidence gaps to inform areas that require further research.

### Review Question

What is the relationship of different groups of prescription medicines with work outcomes in working age people who have sustained injuries or MSDs?

## Methodology

### Inclusion Criteria

The eligibility criteria for inclusion is guided by the Joanna Briggs Institute (JBI) Participant Concept and Context (PCC) mnemonic guide for scoping reviews [30].

#### Participants

This review included studies with participants of working age (18+ years) who experienced injuries or MSDs and received prescription medicine, were dependent on or used medicines following the onset of injury or MSDs. The upper working age is not limited since the aging workforce is increasing, and some countries introduced flexibility in the retirement plan or no mandatory retirement age [31, 32]. However, studies with a population comprised entirely of older adults who are unlikely to be of working age (e.g., 75+ years) were not considered. Injuries or MSDs that occurred in any working age adult were considered broadly, without putting restriction on work-relatedness of injuries/MSDs or receiving treatment under compensation or insurance.

#### Concept

This review considered studies that reported the relationship between medicine use and work-related outcomes. Studies were eligible if medicine use was reported (e.g., prescription opioids, antidepressants, benzodiazepines, NSAIDs, SMRs and other analgesic medicines). Medicine use was broadly defined and described as any payment/reimbursement of prescriptions, dispensing of medicines, direct administration/actual use/consumption of medicines or dependence on prescription medicines (this includes workers with MSDs/injuries who developed prescription opioid dependence and were accessing rehabilitation for opioid dependence as a part of a return to work intervention). Work-related outcomes were broadly defined to include concepts such as return to work, work function, work retention, work loss, work disability, duration of disability, time loss/ time off work, absenteeism, sickness absence, benefit/wage replacement duration, claim closure, indemnity claim cost for lost time and physical function improvements or self-reported functional disability.

#### Context

Studies were included regardless of the type of MSDs or injuries present, as well as whether or not the individual was receiving support from benefit schemes such as workers’ compensation or sickness absence. We only included studies focused on clinical or community settings (i.e., real-world settings) for medicines that had received regulatory approval and were available on the market. Safety and efficacy pre-market studies of phase II and III clinical trials were not considered for inclusion. These studies are in the earlier stages and conducted for regulatory and market approvals for the clinical conditions being studied and were not considered to reflect real-world evidence and practice.

#### Types of Sources

All types of studies except opinion papers, letters, case reports and abstracts/conference proceedings were considered for inclusion in this scoping review. Studies published after 2000 and published only in English language were considered for this review. The time-based limit (year 2000 onwards) was adopted to ensure that the review summarised more contemporary evidence, and to focus on a period during which there has been a growing clinical and regulatory focus on prescription medicine use among injured workers. This reflects the period of time over which the use of opioids for chronic pain (including in injured workers) and opioid-related harms increased dramatically [33, 34]. Studies investigating medicine use for injuries/MSDs due to neoplasms or during pregnancy were excluded.

### Protocol Registration and Deviation from the Protocol

This scoping review was conducted in accordance with a protocol prepared based on JBI methodology for scoping reviews [30]. The protocol was published a priori on the Open Science Framework and is available at https://osf.io/4jxg7. A deviation from the protocol should be noted as the secondary outcome (safety outcome) are not included in this review. The authors considered that the relatively diverse and large number of studies included for the primary outcome would be better addressed independently with this review. The data extraction form was modified from the protocol as expected, capturing some details included for studies on participants who underwent surgical procedures such as the type of surgical procedure, preoperative and postoperative medicine use. The approach to data extraction was also modified for feasibility reasons. Furthermore, one addition in the review is the exclusion of pre-market studies involving Phase II–III safety and efficacy clinical trials, which was not anticipated in the study protocol. This scoping review follows the final reporting guidance recommended by the PRISMA Extension for Scoping Reviews [35].

### Search Strategy

The search strategy followed a three-step approach. The first step was developing a logic grid including preliminary search terms aligned with the scoping review participant, concept, and context. Then, a preliminary search on two databases (MEDLINE and EMBASE) via the Ovid platform was conducted. The search texts and index terms were identified in the preliminary search and used to develop the full search strategy for the MEDLINE database. The full search strategy employed via OVID platform was presented in the supplementary Table 6. In the second step, the search strategy was adapted and replicated in other electronic bibliographic databases. Boolean, Truncation, Wildcard and Proximity operators were employed to increase both the sensitivity and specificity of search results. The third step involved hand searching of reference lists and forward citation searching of included studies. We searched MEDLINE, EMBASE, PsycINFO, CINAHL Plus, Scopus, Web of Science and Cochrane library such as Cochrane Central Register of Controlled Trials (CENTRAL) databases on January 27, 2022. The original search from these databases was updated on June 23, 2023. Grey literature was searched from dissertation, theses, and grey literature databases including the National Institute for Occupational Safety and Health (NIOSH) databases, OpenGrey, The Grey Literature Report (GreyLit Report), National Institute for Health and Care Excellence (NICE) and Turning Research Into Practice (TRIP) databases.

### Study Selection

All final identified records from each database were imported to the Covidence platform (www.covidence.org) to remove duplication and to support article screening. Titles and abstracts were screened by two independent reviewers (YGT and AG). Potentially relevant papers were retrieved for full text review. The full text articles were assessed in detail against the inclusion criteria by two independent reviewers (YGT and AG). Any disagreements between the reviewers at each stage of the selection process were resolved through discussion or with a third reviewer (AC) where necessary.

### Data Extraction

Data were extracted using a data extraction form prepared by the authors. First, two reviewers (YGT & AG) independently extracted four articles and full agreement achieved in the extracted data. Then a single reviewer (YGT) extracted the data of the remaining articles and random checking was conducted by the second reviewer (AG).

### Data Analysis and Presentation

Descriptive numerical summaries of the studies including, study sample, type of injuries and/or MSDs, outcome measure and prescription medicine characteristics were developed. Further, the study designs and settings, data sources of prescription medicine and how the outcome was measured were summarized and discussed amongst the investigators, who agreed on the best approach to data presentation. Details of the included studies and summaries of the findings are provided in the supplementary tables. We present the review findings after grouping the studies with similar MSDs/injury types and combining similar medicine groups (e.g., including duration of use and other common characteristics) to organize the reported relationship with work outcomes. First, grouping of studies were developed by the type and nature of MSDs and injuries. Then, subgroups were constructed based on the types and characteristics of medicine use within the specified group. The direction of the relationship between prescription medicine and work outcomes, the relative homogeneity in exposures such as duration of medicine use and context and setting type were used to guide the evidence narration of the results.

## Results

### Study Inclusion

**Fig. 1:**
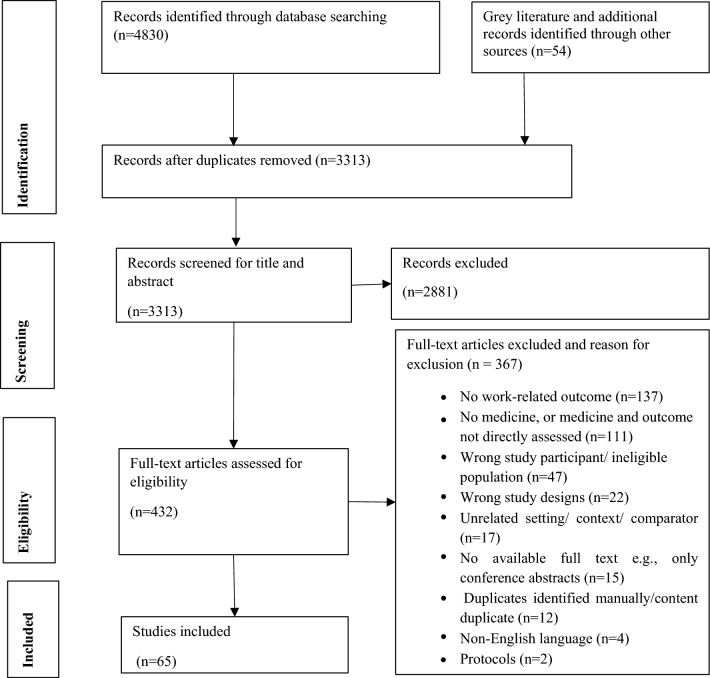
PRISMA Flow chart of article search and screening process

A total of 4884 records were identified through the literature search. After removing duplicates, 3313 articles progressed to title and abstract screening. A total of 432 full text articles were retrieved and assessed for eligibility, with 65 studies included (Fig. 1).

### Characteristics of the Included Studies

A summary of the included studies, including a high-level summary of study findings, is provided in Table 1. Most (*n* = 50) of the studies were conducted in the United States, followed by Australia (*n* = 5), Canada (*n* = 3), Denmark (*n* = 2) and one in each of the following countries: United Kingdom, Netherlands, Ireland, and India. One study was conducted in participants from multiple countries [36]. Most of the studies were cohort studies (*n* = 48) (36 retrospective and 12 prospective cohort studies), followed by randomized controlled trials (RCTs) (*n* = 10), cross-sectional studies (*n* = 3), case series (*n* = 2), case control (*n* = 1) and a single longitudinal ecological study (*n* = 1).

**Table 1:** Overview of included studies and high-level summary of the study findings

MSD/ Injury type	First author, year [citation] and country	Study design	Prescription medicine (exposure)	Setting	Relationship of exposure with work/functional- outcome
Acute low back pain/ injuries	Bernstein (2004) [37] USA	Prospective cohort	SMRs	NB and WC	Negative*
	Friedman (2019) [38] USA	RCT	SMRs (Baclofen, Metaxalone, Tizanidine)	NB	No relationship
	Ralph (2008) [39] USA	RCT	SMRs	NB	Positive*
	Friedman (2015) [40] USA	RCT	SMRs or opioids/acetaminophen added on NSAIDs	NB	No relationship
	Mahmud (2000) [41] USA	Retrospective cohort	Opioids; NSAIDs; SMR; acetaminophen	WC	No difference for Acetaminophen, NSAIDs* Negative for Opioids > 7 days*
	Carnide (2019) [27] Canada	Retrospective cohort	Opioids, NSAIDs, SMR (used within 8-weeks after LBP injury)	WC	Negative for opioids and longer supplies of NSAIDs and SMRS*
	Gaspar (2021) [42] USA	Retrospective cohort	Opioids; NSAIDs; SMR; acetaminophen	WC	Negative for opioids*
	Friedman (2017) [43] USA	RCT	Benzodiazepines/ diazepam	NB	No relationship
	Busse (2015) [44] Canada	Retrospective cohort	Opioids within the first 28 days	WC	Negative*
	Cifuentes (2012) [45] USA	Retrospective cohort	Opioids (shorter time gap between opioid prescriptions)	WC	Negative for longer gap between opioid prescriptions* Positive for shorter days between opioid prescriptions*
	Larson (2018) [46] USA	Retrospective cohort	Opioids; tramadol (in the first 30 days)	MTF	Negative*
	Lee (2016) [47] USA	Retrospective cohort	Opioid (during initial ED visit and filled within 2 days of that visit).	WC	No relationship*
	Webster (2007) [48] USA	Retrospective cohort	Opioids (higher MEA received in the first 15 days postinjury)	WC	Negative for higher dose* No relationship for low dose*
	Franklin (2008) [28] USA	Retrospective cohort	Opioids for > 7 days during the first 6 weeks	WC	Negative*
	Gross (2009) [29] Canada	Retrospective cohort	Opioids (within 2 weeks)	WC	Negative*
Chronic or unspecified low back pain	Urquhart (2018) [49] Australia	RCT	Tricyclic antidepressants Low-dose amitriptyline (25 mg) for 6 months	NB	No relationship at 6 months* Positive at 3 months*
	Phelps (2001) [50] USA	Retrospective cohort	Opioids; NSAIDs; SMRs	WC	No difference (Opioids, NSAIDs and SMRs)
	Chu (2012) [51] USA	RCT	Opioids (Morphine for 1 month)	NB	Positive
	Mayer (2014) [52] USA	Prospective cohort	Opioids (Opioid dependence)	WC	Negative*
	Preuper (2014) [53] Netherlands	RCT	Opioids/acetaminophen	NB	No relationship
	Weil (2017) [54] USA	RCT	Opioids ALO-02 (an abuse-deterrent formulation containing extended-release oxycodone and naltrexone)	NB	Insufficient evidence (No difference )
	Ashworth (2013) [55] UK	Prospective cohort	Opioids (baseline opioid use in the 28 days)	NB	Negative*
	Di Donato (2022) [56] Australia	Retrospective cohort	Opioids (opioid dispensing over 2.5 years)	WC	Negative*
	Volinn (2009) [57] USA	Retrospective cohort	Opioids	WC	Negative*
	Savych (2019) [58] USA	Retrospective cohort	Opioids	WC	Negative for long-term opioid prescriptions* No relationship for shorter-term opioid prescriptions*
Osteoarthritis	Goorman (2000) [59] USA	Case series	3 weekly intra-articular injection of Hylan G-F 20	NB	Positive
	Thakkar (2021) [60] USA	Retrospective cohort	Opioids	WC	Negative*
Other musculoskeletal conditions (injuries such as, fracture, dislocations sprain/strain, multiple body part injuries, shoulder, back, knee, thigh, lower and upper extremity injuries and other non-specified musculoskeletal conditions)	Tillander (2018) [36] Runners from Multiple countries	Cross-sectional	NSAIDs	NB	Positive
	Sundstrup (2017) [61] Denmark	Prospective cohort	Regular use (at least 14 days within the last year)	SAC	Negative*
	Johnston (2016) [62] USA	Retrospective cohort	Opioids	WC	Negative*
	Szucs (2022) [63] Australia	Retrospective cohort	Opioids	WC	Negative*
	Berecki-Gisolf (2013) [64] Australia	Retrospective cohort	Opioids (within 10 days)	WC/ TAC	Negative*
	Haight (2020) [65] USA	Retrospective cohort	Prescribed at least one opioid within the first 6 weeks of injury and high-risk opioid prescribing	WC; SS and DP	Negative*
	Kidner (2009) [66] USA	Prospective cohort	Opioids	WC; SS; SSI	Negative*
	MacLaren (2006) [67] USA	Prospective cohort	Opioids	WC	No relationship
	White (2020) [68] USA	Retrospective cohort	Opioids (Opioid use disorder)	PI/ SI	Negative*
	Hunt (2019) [26] USA	Retrospective cohort	Opioids; benzodiazepine; antidepressant	WC	Negative for antidepressants, opioids, and benzodiazepines*
	Lavin (2014) [17] USA	Retrospective cohort	Opioids; benzodiazepines	WC	Negative for both opioid and benzodiazepines*
	Tao (2015) [69] USA	Retrospective cohort	Opioids: psychotropics: antidepressants; anti-anxiety agents antipsychotics and hypnotics	WC	Negative for all psychotropics, opioids and their combined use*
	Tao (2015) [70] USA	Retrospective cohort	Opioids; antidepressant; hypnotics, antidepressant, or antianxiety agent. Early prescription (initial 60 days)	WC	Negative for all psychotropics*
	Nkyekyer (2018) [71] USA	Cross sectional	Opioids; benzodiazepines Pre-injury opioid or benzodiazepine	WC	Negative
	Brede (2012) [72] USA	Prospective cohort	Opioids	NB; SC and SI	Negative*
	Franklin (2019) [73] USA	Longitudinal ecological	A sustained reduction in persistent opioid use (between 6- and 12-weeks following injury)	WC	Negative for persistent opioid use
	Lavin (2016) [74] USA	Retrospective cohort	Opioids	WC	No difference for opioids less than 30 days* Negative for prolonged opioids prescriptions (mainly for > 180 days)*
	Lavin (2017) [75] USA	Retrospective cohort	Prescription of opioids for 3 or more consecutive months	WC	Insufficient (Inconclusive as argued by the authors)
	Dersh (2008) [76] USA	Prospective cohort	Post-injury prescription opioid dependence	WC	Negative*

Negative outcomes included a slower return to work, longer duration of disability, delayed claim closure, poorer self-reported work function, poorer functional improvement. Positive outcomes included better return to work, shorter duration of disability, shorter wage replacement duration, improved self-reported disability and better functional improvement.

*ALO-02* oxycodone hydrochloride and sequestered naltrexone hydrochloride, *DP* Disability Pension, ED emergency department, *MEA* Morphine Equivalent Amount, *NB* No benefit, *NSAIDs* Non-steroidal Anti-Inflammatory Drugs, *MTF* Military treatment facility, PI*/SI* privately insurance/self-insurance beneficiaries, RCT Randomized control trial, *SAC* Sickness Absence scheme, SI Supplemental income, SS social security, SMR Skeletal Muscle Relaxant, *TAC*  Transport Accident Commission, WC Workers’ compensation

*Adjusted for covariates and potential confounders in multivariable models of statistical analysis

Work related outcomes were obtained from administrative/benefit data in 41 studies followed by self-reported scales (*n* = 21) (including the Roland–Morris Disability Questionnaire (RMDQ) was used in eleven, Oswestry Disability Index and Short Form-36 Health Survey each were each used in three studies), medical records (*n* = 2) and both benefit and self-reported data (*n* = 1). Prescription medicine data were collected from workers’ compensation (WC) data (*n* = 37), linked prescription databases/medical records (*n* = 25) and self-report (*n* = 3).

Most studies (*n* = 40) included samples of workers who were receiving a WC benefit or service. Seven studies included people receiving benefits/services from other income support schemes (such as sickness absence, military, transport accident compensation and private insurance), four studies were in mixed populations of WC, sickness absence and social security schemes. Fourteen of the included studies did not report how the services were funded.

Most of the studies reported opioid use (*n* = 55) followed by NSAIDs (*n* = 8), SMRs (*n* = 8), antidepressants (*n* = 5), benzodiazepines (*n* = 4), acetaminophen (*n* = 3), pregabalin (*n* = 2) and other psychotropic medications (*n* = 2). Most studies were conducted in people with back disorders (*n* = 41), two studies were on shoulder injury (one on rotator cuff injury and one on shoulder impingement syndrome), one on carpal tunnel syndrome, one on lower limb injury, two on osteoarthritis, injuries/musculoskeletal conditions of multiple body parts (*n* = 8) and non-specified musculoskeletal conditions/injuries (*n* = 10).

Nineteen of the studies involved workers with MSDs and injuries who underwent a surgical procedure, mostly spine surgeries of the back (*n* = 15). Of these, thirteen assessed preoperative opioid use and its association with post-operative work outcomes.

### Prescription Medicines Use in MSDs/Injuries of the Back and Its Work Outcome

#### Opioids in Acute/Subacute Low Back Pain

One RCT and ten observational (one prospective and nine retrospective cohort) studies assessed the association of early opioid prescription (i.e., defined as any opioid prescriptions in the first two months of injury/illness onset) [27] with subsequent work-related disability in patients with acute LBP. Nine of these studies were in WC settings [27–29, 41, 42, 44, 45, 47, 48] and one in a military treatment facility [46]. Two studies examined opioid use in the first week of post injury/pain onset [42, 47], two weeks (*n* = 2) [29, 48], four weeks (*n* = 3) [44–46] and greater than 7 days during the first six to eight weeks of injury/LBP onset (*n* = 3) [27, 28, 41].

Eight of the studies reported that early opioid prescriptions were associated with impaired functional recovery and greater risk of work disability while two studies reported no association with work disability (Supplementary Table 1). Early opioid prescriptions within the first [42] or second week of acute injury or LBP onset [29], opioids lasting greater than 7 days [28, 41], or higher dose supply [48]were found to be a predictor of subsequent work disability or delayed recovery. The use of higher morphine equivalent amount (more than 450 mg) were associated with prolonged work disability(on average, 69 days (49.3–89 days) longer disability than WC claimants who received no early opioids) but no difference was observed between those received lower doses (up to 140 mg) and those received no opioids in the first 15 days [48].

Opioid treatments within the first one month of acute LBP were associated with longer claim duration [HR: 0.68 (0.53–0.88)] [44] and greater duty limitation [OR 1.14 (1.04–1.26)] [46]. Likewise, provision of opioids within 8-weeks after LBP/injury were associated with prolonged work disability [10% (1.09–1.11) increase in the number of days on benefit for each 7-day increase in cumulative days supplied for opioids] compared with NSAIDs and SMRs [27]. Workers who received strong opioids had a greater risk of work disability compared with those received only weak opioids [27].

Two studies found that early opioid prescriptions for acute LBP in the emergency department (ED) had relationship with disability duration [47] or adding opioids to naproxen (for 10 day treatment regimen) had no effect on functional outcomes after 1-week of ED discharge [40]. However, in another study, opioid prescriptions during the first month of acute LBP were linked to 14% longer disability for every additional week between prescriptions RR: 1.14 (1.06–1.22), and fewer days between opioid prescriptions were associated with shorter time off work [45].

#### Skeletal Muscle Relaxants, Benzodiazepines and NSAIDs in Acute/Subacute Low Back Pain

Seven studies examined the association between SMRs prescriptions and work disability in patients with acute or subacute LBP. The results were conflicting; two cohort studies reported that SMRs were associated with delayed functional recovery [HR: 0.81 (0.69–0.94)] [37] and that longer supply is associated with greater work disability [with each 7-day increase in cumulative days supplied for SMRs within 8-weeks, the increase in the incidence rate ratios (IRR) of days on disability benefit were 1.03 (1.011.04)] [27], and three studies (two RCT and one cohort) found no difference in self-reported disability [mean improvement in RMDQ score after 1-week: Ibuprofen+Placebo, 11.1 (9.0–13.3); Ibuprofen+Baclofen, 10.6 (8.6–12.7); Ibuprofen+Metaxalone, 10.1 (8.0–12.3); Ibuprofen+Tizanidine, 11.2 (9.2–13.2) [38], Naproxen+Placebo, 9.8 (7.9–11.7) vs. Naproxen+cyclobenzaprine, 10.1 (7.9–12.3) [40] or length of disability (19 days vs. 17 days, *p* = 0.269) [41] between SMRs and other treatment alternatives such as NSAIDs/with placebo. Two further studies (one RCT and one cohort) indicated positive outcomes of SMRs use on functional improvement [improvement in RMDQ score at both Day 3 (6.9 vs. 8.7) and Day 7 (4.1 vs. 6.2), *p* < 0.0001] [39] and returned to work after injury in fewer days, 11.5 days; 95% CI: (− 13.9, − 9.1) [42].

Four retrospective cohort studies on NSAIDs and non-opioid analgesics [27, 29, 41, 42] and one RCT study on benzodiazepine prescriptions [43] assessed the relationship with work disability in people with acute LBP. One study showed neither acetaminophen nor NSAIDs were associated with length of disability [41] while another study found longer supply of NSAIDs in the first 8 weeks were associated with greater work disability [27]. Workers’ compensation claimants who received early non-opioid analgesia experienced delayed recovery compared with claimants who did not receive analgesia [29]. Another study showed that workers who received NSAIDs missed fewer workdays than workers who received opioids [42]. In a study comparing benzodiazepine use for acute LBP to placebo, diazepam use was not associated with functional improvement seven days after the emergency department presentation [43] (Supplementary Table 1).

#### Opioids in Chronic Low Back Pain

Nine studies examine opioid use in people with chronic LBP, with five reporting worse work-related outcomes [52, 55, 56, 57, 58], three reporting no difference [50, 53, 54] and one reporting that opioids were associated with improved outcomes [51]. (Supplementary Table 2).

Two prospective cohort studies reported a negative relationship between opioid use and work outcomes in chronic LBP: one showed opioid prescription at baseline predicted higher disability after six months [55], and the other one reported that post-injury opioid dependence predicted higher disability [two times lower rates of return to work (RTW) and work retention] at one year [52]. Three retrospective cohort studies also observed that work disability markedly increases as duration and strength of opioid increases [56–58]. Among WC claimants who filled opioid prescriptions over an extended period (≥ 90 days), the odds of chronic work loss were almost 11 times among those who received weak opioids (*OR* = 10.9) and more than 14 times greater for those who received strong opioids (*OR* = 14.2) in comparison to those who did not receive opioids. Regardless of the duration of opioid prescribing, chronic work loss was almost twice (*OR* = 1.9) and six times (*OR* = 6.1) higher for those who filled weak and strong opioids prescriptions, respectively, when compared to those without any opioid prescriptions [57]. In another study, longer-term opioid prescriptions roughly tripled the duration of temporary disability, compared to workers with similar injuries who did not receive opioids or longer-term prescriptions. However, no evidence was observed of a causal effect of shorter-term opioid prescriptions in prolonging the duration of temporary disability [58].

One observational study found no difference in RTW between people receiving NSAIDs or narcotics [50] while findings from three RCTs were inconsistent. One observed no significant difference in functional capacity or self-reported disability between the groups receiving tramadol/acetaminophen or placebo [53]. Another observed a reduced activity impairment in patients treated with an abuse-deterrent formulation containing oxycodone–naloxone compared with placebo but without significant difference in working time loss between groups [54]. Finally, one double blinded RCT observed that patients treated with morphine for 1 month experienced clinically relevant improvement in functional ability compared to patients who received placebo [51].

#### Antidepressants, NSAIDs and SMRs in Chronic Low Back Pain

There were two studies in this sub-group. A double blinded RCT of low dose (25 mg) amitriptyline vs. an active comparator reported no difference in work absences OR; 1.51 (0.43–5.38) or hinderance OR, 0.53 (0.19–1.51) and disability at 6 months [adjusted difference in RMDQ score, 0.98 (2.42 to 0.46)] but a decrease of disability at 3 months [adjusted difference in RMDQ score, 1.62 (2.88 to 0.36)] [49]. Another retrospective cohort study showed both NSAIDs (*p* = 0.45) and SMRs (*p* = 0.11)were not related with the probability of successful RTW [50] (Supplementary Table 2).

#### Preoperative Opioid Use and Post-operative Work Outcomes

These studies included any opioid prescribed prior to surgery as a part of pain management. In most cases, this involves chronic use as demonstrated in the included studies. Among the 13 studies on preoperative opioid use, 12 were conducted in patients undergoing spinal surgery [77–88] and one in patients undergoing Carpal tunnel release surgery [89] (Table 2). Eleven studies (all in WC settings) found preoperative opioid use was associated with poor post-surgery work outcomes [77–83, 85–87, 89]. One study (in non-WC setting) showed no association between preoperative opioid use and post-surgery work outcomes [84]. Similarly, another study in a non-WC setting showed persistent opioid prescription were associated with poor postoperative work outcomes while people with recently initiated opioids reported better outcomes. Patients with long-term and high-dose preoperative opioid use experienced the poorest postoperative work outcomes [88] (Supplementary Table 3).

**Table 2:** Pre-, post or perioperative medicine use and postoperative work outcomes in patients with MSDs/injuries undergoing surgery

Study and country	Surgery	MSD/ injury type	Prescription medicine	Work outcome relationship
Anderson (2015) [77] USA^*WC*^	Discogenic fusion	Degenerative disk disease (DDD)	Preoperative opioid use > 1 year	Negative*
Anderson (2016) [78] USA^*WC*^	Lumbar fusion	Spondylolisthesis	Preoperative opioid use > 1 year	Negative*
Anderson (2018) [79] USA^*WC*^		DDD	Preoperative opioid use > 1 year	Negative*
Zakaria (2020) [88] USA^*NB*^		Back pain (spondylosis and intervertebral disc disorders)	Preoperative opioids New opioid users (< 6 weeks) and chronic opioid users (> 6 months)	Positive for new opioid users* Negative for chronic opioid users*
McMillan (2022) [85] Australia^*WC*^		Non-catastrophic workplace injury	Preoperative opioid use	Negative*
Faour (2017) [81] USA^*WC*^	Multilevel cervical fusion	DDD or Radiculopathy	Preoperative opioid prescriptions	Negative*
Faour (2017) [82] USA^*WC*^	Single-level cervical fusion	Cervical Radiculopathy	Preoperative opioids use up to > 6 months	Negative*
Faour (2018) [83] USA^*WC*^			Preoperative opioid use	Negative*
Faour (2017) [80] USA^*WC*^		DDD	Preoperative opioid use	Negative*
Hills (2019) [84] USA ^*NB/medicaid/private insurance^	Spine surgery	Back pain (cervical and lumbar)	Preoperative chronic opioids use (3 months duration)	No relationship*
O’Donnell (2018) [86] USA^*WC*^	Lumbar discectomy (single level)	Lumbar disk herniation (LDH)	Preoperative opioid use	Negative*
Tye (2017) [87] USA^*WC*^	Lumbar decompression	Degenerative lumbar stenosis	Preoperative opioid (greater than 3 months of opioid use)	Negative*
Kho (2017) [89] USA^*WC*^	Carpal tunnel release (CTR) surgery	Carpal tunnel syndrome	Preoperative opioids	Negative
Anderson (2015) [90] USA^*WC*^	Lumbar fusion	DDD and discogenic LBP	Postoperative opioid use	Negative*
Burke (2010) [91] Ireland^*NB*^	Lumbar discectomy	Chronic LBP	Perioperative pregabalin use	Positive
Khurana (2014) [92] India^*NB*^			Perioperative pregabalin use	Positive
Webster (2004) [93] USA^*WC*^	Lumbar intradiscal electrothermal therapy procedure (IDET)	Low back pain	Pre and post procedure opioids	Negative for opioid use before IDET*
Rudbeck (2013) [94] Denmark ^*NB; WC; SAS, UB*^	Arthroscopic subacromial decompression (ASD)	Shoulder impingement syndrome	Perioperative opioids; NSAIDs; antidepressant; acetaminophen	Negative for Opioid /strong pain killers during first year after ASD* No relationship for medication use before ASD (6 months)*
Kraus (2021) [95] USA^*NB and WC*^	Arthroscopic rotator cuff repair	Rotator cuff injury	Post-operative opioids; NSAIDs	No differences for ibuprofen and opioids*

*NB* No Benefit Scheme, *SAS* Sickness Absence Scheme, *UB* Unemployment Benefit, *WC* Workers’ Compensation

*Adjusted for covariates and potential confounders in multivariable models of statistical analysis

Nine of the studies were retrospective cohort or case control studies conducted in the state of Ohio, USA which used WC claims data to examine the relationship between preoperative opioid use and post-operative RTW. In these studies, stable RTW was defined if the worker returned to work and maintained continuous at-work status for at least 6 months within a 3-year period after surgery. In all of the studies, preoperative opioid use in patients who underwent cervical or lumbar surgeries was  observed to be associated with less stable RTW after surgery. The findings were consistent regardless of the duration of use including patients with preoperative opioid use for more than 3 months or 1 year [77–83, 86, 87]. Similarly, in an Australian retrospective cohort study using WC claims data, preoperative opioid use was associated with having only partial or no work capacity at 24 months after surgery and the odds of this outcome were 3 times higher with higher opioid doses (> 40 mg/day) [85]. In contrast, one prospective cohort study of spine surgery registries in a non-WC setting showed that preoperative new opioid users (< 6 weeks) were more likely and long-term users (> 6 months) less likely to have improved functional outcomes 2 years after surgery [88]. Unlike the previous studies, a prospective cohort study of linked data of spine registries in non-WC setting demonstrated long-term preoperative opioid therapy (for 3 months and above) was not associated with RTW status but associated with less improvement in patient reported function (i.e., less than 30% clinical improvement) at 1 year post-surgery [84].

#### Peri- or Post-operative/Procedure Medicine Use and Work Outcomes

Five studies assessed peri- or post-operative prescription medicine use in patients with lumbar [90–92], arthroscopic rotator cuff repair [95] and arthroscopic subacromial decompression surgeries [94], and one further study assessed pre- and post-procedure opioid use in LBP patients received lumbar intradiscal electrothermal therapy [93]. These studies were conducted in WC [90, 93], non-WC [91, 92] or mixed population of both WC and non-WC settings [94, 95] (Supplementary Table 4).

Two small RCTs in patients with chronic LBP in India (*n* = 90) and Ireland (*n* = 38) showed that perioperative pregabalin use was associated with an improvement in functional outcomes 3 months after lumbar discectomy when compared with placebo [91, 92]. There were inconsistent findings among four studies on peri- or post-operative use of analgesics such as opioids and NSAIDs. In a retrospective cohort study of WC records of patients with lumbar fusion, post-operative long-term opioid therapy (> 1 year duration) was negatively associated with RTW when compared with opioid therapy for less than 1 year [*OR* = 0.38 (0.25–0.57)] [90].

In comparison, a prospective cohort study in patients with rotator cuff repair demonstrated no difference in self-reported functional outcomes at 1 and 2 years after surgery between those prescribed post-operative opioids and Ibuprofen [95]. Another retrospective cohort study of medicine use in arthroscopic subacromial decompression surgery showed that using painkillers, particularly strong painkillers/opioids, during the first year following surgery was associated with long-term sick leave [*OR*; 3.78 (2.32–6.16)] and permanent benefits [*OR*; 24.80 (7.05–87.18)]. However, medicine use within 6 months prior to surgery was not associated with these outcomes [94]. Finally, one case series study reported opioid use for three months in LBP patients before lumbar intradiscal electrothermal therapy was a risk factor for poor RTW outcomes [*OR*; 0.20 (0.07–0.57)] [93].

### Prescription medicines Use in Other Musculoskeletal Conditions and Work Outcomes

#### Opioids, Others Psychotropics and Their Combination in Workers’ Compensation Settings

Sixteen studies showed that the use of opioids and psychotropic medicines in workers with musculoskeletal conditions was associated with adverse work-related outcomes [17, 26, 60, 62–66, 68–74, 76]. This was more apparent when psychotropic medicines were prescribed in combination and used with opioids (Supplementary Table 5). Four studies demonstrated that the combined use of opioids and psychotropics was associated with increased claim costs and prolonged work disability [17, 26, 69, 70].

In a retrospective cohort study of WC claims, being on antidepressants [*OR*: 2.24 (2.00–2.51)] were found to be strongly associated with poor RTW (delayed claim closure) in comparison to opioids [*OR*: 1.14 (1.02–1.27)] and benzodiazepines [*OR*: 1.38 (1.23–1.54)]. The addition of anti-depressants to opioids significantly associated with increased delayed claim closure [26]. Similarly, the addition of benzodiazepine to opioid treatment substantially increased the WC indemnity claims cost of lost time (> USD $100,000), *OR*: 2.74 [2.31–3.26] [17]. Two other retrospective cohort studies showed combined use of psychotropics (i.e., any hypnotics, antipsychotics, antianxiety agents, antidepressants) and opioids was associated with poor RTW and increased claim costs [69, 70]. The prescription of long-acting opioids during the first 60 days after injury was found to be the most important indicator of high claim cost and longer claim duration [70]. Another study which assessed prescription medicine use 12 months before injury and 90 days after injury found that pre-injury opioid or benzodiazepine use was associated with a higher prevalence of compensable claims compared to no pre-injury opioid (28.6% vs. 19.5%) or benzodiazepine use (29.7% vs. 20.0%) [71].

One prospective and four retrospective cohort studies in people with MSDs reported either high dose, strong or long-term opioid prescriptions were associated with increased lost workdays, including a study on patients admitted to interdisciplinary functional restoration [66], employed workers with osteoarthritis [60] and WC claimants [65, 74] and a study of workers with lower limb injury [63]. Similarly, a retrospective cohort study on persons injured with motor vehicle accident also reported early opioid prescriptions (within 10 days of accident) were a risk factor for work disability ≥ 6 months after the accident [64]. However, one study showed that high claim cost (at least USD $100,000) and long-lost time duration (at least 3 years) were not statistically different between groups prescribed opioids less than 30 days and nonopioids. But claim cost and lost time were associated with prolonged opioid prescriptions continued after 180 days [74]. In contrast, a prospective cohort study of patients who completed a multidisciplinary rehabilitation showed no significant differences on RTW outcomes at 6 months of post-treatment between those who used opioids and nonopioids [67].

Post-injury dependence on opioid medications were associated with poor work-related outcomes. Two prospective cohort studies of patients referred to a rehabilitation center showed that opioid dependence predicted failure to RTW and retain work at 1 year after functional restoration treatment [72, 76]. Similarly, total lost workdays, lost wages and total healthcare costs were significantly higher in employees with opioid use disorders [62, 68]. Following the implementation of limiting opioid use policy to reverse the opioid crisis in Washington state, reduction of chronic opioid use was associated with the reversal of the increased lost work time patterns observed in previous years [73].

#### NSAIDs and Other Medications

A case series of three weekly intra-articular injection of Hylan G–F 20 for knee Osteoarthritis six months after injection demonstrate overall functional improvement [59]. A cross-sectional survey of professional runners from multiple countries registered for marathon found runners stated habitual NSAID use in the past 12 months reported fewer time-loss injuries (avoidance of running for more than 3 weeks because of injury) than non-NSAID users [36]. In contrast, in a study of employed workers in Denmark reported regular use of pain medication (for at least 14 days within the last year either over-the-counter or doctor prescribed) due to musculoskeletal pain was prospectively associated with long-term sickness absence (at least 6 consecutive weeks). This association was stronger with regular use of doctor prescribed pain medication *HR*: 2.18 (1.67–2.86) when compared with the use of over-the-counter pain medication *HR*: 1.44 (1.13–1.83) [61].

## Discussion

This scoping review aimed to explore the available evidence on the relationship between prescription medicine use and work outcomes in people with MSDs or injury. The review identified that most peer-reviewed literature reports an association between work function impairment and opioids and other medicines prescribed in working age people with MSDs or injury. This was more evident with early initiation of opioid prescriptions, long-term use, strong and/or high dose of opioids, extended pre-and post-operative opioid use in elective surgeries and psychotropic medicines in WC setting. The relationship between prescription medicine and adverse work outcomes requires cautious interpretation as the vast majority of evidence is from cohort studies demonstrating an association, and there have been relatively few RCTs which may provide more direct causal evidence. The relationships observed might be partly caused by confounding by indication or residual confounding such as baseline pain and injury severity, though most observational studies included adjustment for multiple confounders. A total of 49 studies (47 of them are observational studies) adjusted potential confounders and covariates in the statistical analysis (mainly used multivariable regression models). Forty-four, eight and three studies reported negative, no and positive relationship between prescription medicines and work outcomes, respectively. The covariates and potential confounding variables adjusted in majority of studies included sociodemographic, baseline pain type and injury, duration of pain and severity of injury, psychiatric and other comorbidities, other health service use such as physiotherapy and chiropractic care, pre-/surgery related information, workplace and compensation related factors such as attorney involvement and prior claim history. This may indicate that prescription medicines are independently statistically associated with work outcomes. But the set of co-variates included in each multivariable model vary from study to study. The extant evidence base is highly concentrated on opioid use, however adverse work outcomes were also observed in people using other groups of medicines routinely utilized to assist recovery and relieve pain such as NSAIDs and SMRs. The relationship between medicine use and adverse work outcomes was also observed across countries and systems of work disability support, and in studies that assessed work function using a variety of methods. This review adds to the substantial evidence of the potential adverse safety impacts of medicines such as adverse events and risk of addiction, by examining the important functional outcome of engagement in work. The review findings may provide insights on potential long-term outcomes of prescription medicines beyond their clinical use to relieve pain and symptoms. Our findings suggest that the potential adverse association with the ability to work should be considered during the decision making to prescribe medicines for MSD or injury in addition to the shorter-term clinical effects.

Most of the included studies examined the relationship between opioids and work outcomes and found a negative association. Opioids are intended to be used by people with severe pain and injury when pain control is not attained with commonly used less potent analgesics [11, 12, 14]. However, opioids are often prescribed inappropriately [15] for people with less severe pain and widely used against clinical guidelines for prolonged period [12, 16, 96]. If pharmacotherapy is needed, acetaminophen and NSAIDs are recommended first line medicines [11–14, 97]. Few studies have investigated these commonly prescribed analgesic medicines and its impact on work-related outcomes.

One of the critical findings revealed in this review was the negative relationship between other prescription medicines (such as NSAIDs and SMRs) and work-related outcomes which are routinely prescribed for musculoskeletal conditions. This finding suggests that the use of these medicines for temporary relief of pain might be revisited, given the potential that they may adversely impact long-term work outcomes. However, the evidence is less settled and conflicting results was demonstrated by different studies. The negative relationship is shown only in the observational studies while the RCTs observed either no or a positive relationship between the prescribed medicine and outcomes. This emphasises the need of more higher quality evidence. From the seven studies that assessed SMRs in acute LBP, two identified the direction of relationship with work outcomes as negative [27, 37], three as no relationship [38, 40, 41] and two as positive [39, 42]. Similarly, NSAIDs use in acute LBP has been associated with either prolonged [27], reduced [42], or nonexistent work disability relationship [41]. More evidence is needed because these medicines are increasingly commonly used. Thus, more robust evidence is required from studies with strong study designs such as double blinded RCTs that can control bias and confounding arising from differences in baseline patient characteristics such as pain/injury severity as well as other socioeconomic determinants.

Studies that assessed prescription medicines in WC settings have reported consistent adverse work outcomes across all medicine categories than studies in other settings. All studies in WC settings showed preoperative opioid use was a predictor of poor work outcomes after surgery. This may indicate that frequent opioid prescribing prior to surgery in injured workers could be a red flag for post-operative work outcomes. But one study in a non-WC setting showed no association between preoperative opioid use and post-operative work outcomes [84]. This emphasizes prescribing opioids in WC setting may require more attention than the general setting. This will be informative to revisit funded prescriptions in WC setting to reduce work disability and associated costs for both injured workers and systems that insure them.

### Evidence Gaps

The evidence is accumulated mainly on opioids and is predominantly from observational studies, with a lack of higher quality evidence from RCTs. Evidence is scarce for other psychotropic medicines (e.g., antidepressants, benzodiazepines, and anticonvulsants) despite their increasing use in MSDs and injuries. The extent of preinjury use of these psychoactive medicines which have a potential to increase injury vulnerability, post-injury medicine use and work disability were less commonly investigated. Literature is also limited on potentially inappropriate medicine use indicators such as high-risk opioid prescribing during the earlier phases of injury/illness and the relationship with work outcomes. Further, there is little evidence on polypharmacy and its association with work disability despite the growth of concurrent prescribing in workers’ compensation [98]. Polypharmacy is an important indicator for the number and volume of medicine use. It is also an important predictor for potentially inappropriate medicine use [99] and adverse patient outcomes in other clinical conditions [100]. Clinical audit and evaluation of prescribed medicines concordance to clinical treatment guidelines is also required to better understand the extent of guideline adherence. These would help to determine whether workers are receiving treatments as per best clinical practice recommendations or if adverse work outcomes are persistent regardless of guideline recommendations.

There are interesting findings on pre-, peri- and post-operative opioid use and subsequent post-operative work disability. It would be important to determine to what extent prior medication use and early medication use is an indicator of injury and pain severity versus an independent predictor of outcomes. Conflicting findings with other medications including SMRs and NSAIDs also warrant further exploration.

Multiple aspects of the existing literature may suggest a causal relationship between opioid use and adverse work outcomes. Many (46 out of 55) studies demonstrate a negative relationship, there is a high degree of consistency in the results of these studies, and there are plausible causal mechanisms. For example, prolonged supply of opioids might place workers at risk of misuse and addiction which can inadvertently impact productivity and work outcomes [101]. Additionally, it may cause hyperalgesia, tolerance and dependence which may ultimately distort injured workers future outcome trajectory. These patterns combined with prior research suggest that it is plausible that opioid use is a contributor to work disability among injured workers. There remains a need, however, for more robustly designed studies to confirm this hypothesis. Future studies should seek to clearly differentiate between the impacts of opioids and the underlying clinical, economic, psychosocial, and occupational determinants of work outcomes. Prospective study designs which can control these underlying characteristics are required. Lack of qualitative studies that explore multiple factors associated with medicine use and work outcomes is a further evidence gap. Qualitative studies can accompany prospective studies to explore the attributes associated with post-opioid prescriptions and work disability in injured workers. This may provide greater understanding of the interaction of biopsychosocial factors on the relationship between opioid use and work outcomes. Furthermore, the existing studies are limited to few countries and evidence is lacking notably from developing nations.

### Strength and Limitations

One major strength of this review is the a priori published protocol which enhances transparency. We also employed a comprehensive search strategy addressing medicine use in people with MSDs and injury without restricting setting, medicine type and condition was conducted. This allowed us to explore the available evidence and gaps on prescription medicines and work outcomes. However, the review has the following limitations inherent to the scoping review approach and shortcomings of the existing literature. Scoping reviews do not conventionally provide quality appraisal and graded evidence recommendation. In this review, the heterogeneity of the studies, the variation of study contexts, range of included MSDs and injuries, broad scope of the review and variability in both exposure and outcome measurement made both evidence synthesis and comparison between groups of studies challenging. It is clear that some of the available studies have methodological limitations. Despite the attempts to control the confounding by adjusting in multivariable analysis and limiting the cohorts with comparable MSDs, potential risk of bias (including temporality of the relationship, exposure measurement, selection, and immortal time bias) and residual confounding (e.g., pain and injury severity) is not entirely avoidable as the majority of studies utilized retrospective data such as that collected for administrative purposes. This suggest a need to more strong evidence on adverse outcomes whether independently arise from medicines or an indicator of unobserved characteristics as well as it requires ruling out the association arise from the indication where the medication is prescribed. Thus, clear distinction is required between medicine impacts and the underlying clinical, economical, psychosocial, and occupational determinants of work-related outcomes.

Difference in the chronicity of case and parameter definitions such as opioid use duration (short term, intermediate and long-term opioid use) vary across some of the included studies. Furthermore, characteristics of medicines such as dose, duration of use, and timing of the studies was limited. Most of the studies used WC administrative payment data as the data source, rather than actual medicine consumption. Lower concordance was observed between self-reported and administrative claim or prescription record for medicines used as needed (such as analgesics, hypnotics and sedatives) than other prescription medicines [102, 103]. This shows prescription records (either medicine prescription or dispensation) may not show medicine consumption. However, it is still considered reliable data sources for historical data than self-reported medicine use as it provides more consistency  and avoids recall bias [104].

### Implication for Policy and Practice

This scoping review provides a systematic summary of the existing evidence about the relationship between broad range of prescription medicines and work outcomes in people with MSDs and injuries. Prescription medicine use has been mostly reported to be associated with higher work disability outcomes in working age people with musculoskeletal conditions. These findings may require cautious interpretation and should not preclude medicine use entirely as they are indicated primarily to improve the clinical conditions and alleviate pain of injured or ill patients. However, the findings cannot be disregarded because of the consistency of epidemiological reports and growing concerns that medicine misuse poses risk of preventable harms [105]. These preventable harms were more apparent in funded health services (e.g., WC settings) for prescription medicines with misuse potential such as opioids and other psychotropics. Rational medicine prescriptions guided by the recommended clinical treatment guidelines is required to ensure the appropriate use and reduction of preventable adverse outcomes associated with medicines. Thus, enforcing/ensuring clinicians adherence to recommended therapeutic guidelines (such as LBP guidelines which limit opioids for short term use and appropriate indication) would have paramount importance. Further monitoring systems may be required to ensure the safe and appropriate prescribing and dispensing of high-risk medicines e.g. prescription monitoring programs [106, 107] and prior authorization of opioids use beyond 6 weeks in state of Washington, USA [108] were introduced to prevent harms associated with high risk medicines. The later averted the chronic opioid use and its associated work disability in Washington state workers’ compensation [73].

## Conclusion

Most studies showed a negative relationship between prescription medicine use and work outcomes among working age adults with MSDs or injury. This finding extends current understanding of the impacts of medicine use in worker populations for which prior reviews have focused on side effects, pain, adverse events and other outcomes. Opioids were the most common medicine reported to be associated with adverse work outcomes across various MSDs and injuries. However, the potential causal relationship and mechanisms underpinning this relationship are not well explored. There is also emerging evidence that SMRs and NSAIDs may be associated with adverse work outcomes. However, the evidence on this relationship is conflicting. There remains a need for studies with more robust design to enable more definitive exploration of causal relationships. In the meantime, care should be taken when prescribing medicines to workers with MSD or injury given the potential long-term impacts on ability to engage in work.

## Supplementary Information

Below is the link to the electronic supplementary material.

Supplementary material 1 (DOCX 81.4 kb)

## Data Availability

The data that supports the findings in this review are attached as supplementary files.
