# Supplementary material for: Impact of Prescription Medicines on Work-Related Outcomes in Workers with Musculoskeletal Disorders or Injuries: A Systematic Scoping Review
Source: J Occup Rehabil. 2023 Nov 7;34(2):398–414. doi: 10.1007/s10926-023-10138-y (PMC11180015; doi:10.1007/s10926-023-10138-y)
Supplement: Supplementary file 1 — Supplementary material 1 (DOCX 81.4 kb) [file 10926_2023_10138_MOESM1_ESM.docx]

**Supplementary Table 1: Prescription medicine use in acute and subacute low back pain and its impact on work related outcomes**

| **Study & country** | **Method**   - **Study design,** - **Data collection/source** - **Follow-up and** - **Data analysis** | **MSD/ Injury type** | **Participants characteristics (Sample, Female, Mean age in years)** | **Prescription**  **Characteristics/exposure (group, duration)** | **Setting** | **Work outcome/summary of findings** |
| --- | --- | --- | --- | --- | --- | --- |
| Bernstein 2004  [37]  USA | - Prospective Cohort study - Secondary data analysis of patients and interviewed with RMDQ at baseline, 2, 4, 8, 12, and 24 weeks. - 24 weeks - Cox proportional hazard model | Acute LBP | Muscle Relaxant  n=769  F= 55%  Age: 40.6  No muscle Relaxant  n= 811  F=50%  Age: 42.3 | Skeletal Muscle relaxants | NB & WC | The hazard ratio (HR) to functional recovery for muscle relaxants was 0.81 (0.69-0.94). |
| Friedman 2019  [38]  USA | - Randomized controlled trial - The RMDQ was used to measure LBP functional impairment at baseline and after 1-week. - 1 week - Descriptive statistics | Acute LBP (acute, non-radicular, nontraumatic) | Ibuprofen + Placebo  n=80  F= 36(45%)  Age= 39 ±11  Ibuprofen + Baclofen  n=80  F= 23(29%)  Age= 39±12  Ibuprofen + Metaxalone  n=80  F= 36(45%)  Age= 37±10  Ibuprofen + Tizanidine  n=80  F= 38(48%)  Age= 40±11 | Skeletal Muscle relaxants  - Control arm received ibuprofen 600 mg + placebo orally every 8 hours as needed  - Baclofen arm received ibuprofen 600 mg + baclofen 10 to 20 mg orally every 8 hours as needed  - Metaxalone arm received ibuprofen 600 mg + metaxalone 400 to 800 mg orally every 8 hours as needed, and  - Tizanidine arm received ibuprofen 600 mg + tizanidine 2 to 4 mg orally every 8 hours as needed | NB | Compared with ibuprofen plus placebo, adding baclofen, metaxalone, or tizanidine to ibuprofen does not improve functioning or pain by 1 week after an ED visit for acute low back pain. |
| Ralph 2008[39]  USA | - Randomized controlled trial - Patient completed a functional assessment evaluation using the RMDQ before and after taking medication - 7 days - ANCOVA with fixed effect | Acute lower-back spasm | Carisoprodol  n=269  F= 131 (48.7%)  Age= 39.3 ± 11.82  Placebo  n=278  F= 153 (55.0%)  Age= 41.5 ± 11.7 | Skeletal Muscle relaxants  Carisoprodol 250-mg tablets | NB | The mean RMDQ score at baseline was 10.4 in the carisoprodol group and 10.3 in the placebo group.  A significantly (p < 0.0001) greater improvement in RMDQ score was observed in the carisoprodol group than in the placebo group at both Day 3 (6.9 vs. 8.7) and Day 7 (4.1 vs. 6.2). |
| Friedman 2015 [40]  USA | - Randomized controlled trial - The RMDQ was used to measure LBP functional impairment at baseline ED discharge and after 1-week. - 1 week - Descriptive statistics (intention-to-treat analysis) | Acute LBP (acute, non-radicular, nontraumatic) | Naproxen +  Placebo  n=107  F=53(50%)  Age=39±11  Naproxen +  Cyclobenzaprine  n=108  F=45(42%)  Age=38±11  Naproxen +  Oxycodone/  Acetaminophen  n=108  F=60(56%)  Age=39±11 | A 10-day treatment with 20 Naproxen 500mg tablets (a tablet twice a day) and 60 tablets of placebo; cyclobenzaprine 5mg; or oxycodone 5mg/ acetaminophen 325mg (Participants were instructed to take 1 or 2  of these tablets every 8 hours. | NB | After 1-week follow-up, the mean RMDQ improvement was 9.8 in the placebo group, 10.1 in the cyclobenzaprine group, and 11.1 in the oxycodone/acetaminophen group. The between-group comparisons found no significant difference in mean RMDQ improvement between cyclobenzaprine and placebo, 0.3 (98.3% CI, −2.6 to 3.2; P =0 .77), oxycodone/acetaminophen vs placebo, 1.3 (98.3%CI, −1.5 to 4.1; P = 0.28), and oxycodone/acetaminophen vs cyclobenzaprine, 0.9 (98.3%CI, −2.1 to 3.9; P =0.45).  Adding cyclobenzaprine or oxycodone/acetaminophen to naproxen alone did not improve functional outcomes at 1-week follow-up after ED discharge. |
| Mahmud 2000 [41]  USA | - Retrospective Cohort study: - WC claims filed in 1995. - 1 year - Multivariate Cox proportional hazard model | Acute uncomplicated Low Back Pain | n=98  F=28(28.6%)  Age= median age 34 | Opioids; NSAIDs; Skeletal Muscle relaxants; Acetaminophen/Paracetamol | WC | Neither Acetaminophen, NSAIDs nor muscle relaxants seemed to influence the length of disability (LOD). Patients receiving opioids for greater than 7 days experienced increased LOD compared with patients receiving either no opioid prescription (45 vs 17 days, P=0.036) or opioid prescription for 7 or fewer days (13 vs 17 days, P= 0.074). |
| Carnide 2019 [27]  Canada | - Retrospective Cohort study - Linked compensation, dispensing and healthcare data of workers between 1998 and 2009 with WorkSafe BC. - 1 year - Zero-inflated negative binomial model | Low back pain (Acute and Subacute) | NSAIDs and/or SMRs  n=29104  F= 11313 (38.9%)  Opioids only n=7730  F= 2801 (36.2%)  Opioids with NSAIDs and/or SMRs  n=18737  F= 6403 (34.2%) | Opioids, NSAIDs, Skeletal Muscle relaxants used within 8-weeks after a work-related low back pain (LBP) injury  Work disability = Days on short-term disability benefits (at least for one day) after 8 weeks up to 52 weeks | WC | Provision of early opioids leads to prolonged work disability compared with NSAIDs and SMRs, though longer supplies of all drug classes are also associated with work disability.  Workers receiving strong opioids also had a greater risk of work disability compared with those receiving only weak opioids. |
| Gaspar 2021  [42]  USA | - Retrospective Cohort study: - California's WC claims data from May 2009 to May 2018 were compared with guidelines. - 1 year - Quantile regressions. | Acute uncomplicated LBP | n=59656  F=19,991 (33.5%)  Age=41±12 | Opioids; NSAIDs; Skeletal Muscle relaxants; Acetaminophen  Guideline adherence is a proxy measure of opioid use in the first week.  NSAIDs (strongly recommended), muscle relaxants (moderately recommended), and opioids (strongly not recommended). | WC | Workers prescribed an opioid were typically out of work four days longer than workers not prescribed opioids (medians = 30 vs. 26 days) (p-value <0.001).    Workers who received only guideline-recommended interventions experienced significantly fewer lost workdays (11.5 days; 95% CI: -13.9, -9.1), a 29.3% reduction than workers who received only non-recommended interventions. |
| Friedman 2017  [43]  USA | - Randomized controlled trial - RMDQ used to measure functional impairment before and after the intervention. - 1 week and 3 months after ED discharge - Change in RMDQ score | Acute, nontraumatic, non-radicular LBP | Naproxen + Diazepam  n= 57  F= 30(53%)  Age= 34 ± 12  Naproxen + Placebo  n= 57  F= 24(42%)  Age= 38 ± 12 | Benzodiazepines | NB | One week after the ED visit, patients randomized to diazepam improved by a mean of 11 (9 to 13) RMDQ points, whereas placebo patients improved by 11 (8 to 13) (95%CI for the mean difference of 0.3: (2.8 to 3.5). The between-group difference achieved neither clinical nor statistical significance. |
| Busse 2015  [44]  Canada | - Retrospective cohort study - Workers receiving Ontario workers full benefits at 4 weeks - 2 years - Cox proportional hazards regression | acute LBP (strain/sprain) | n=1442  F= 552 (38.3%)  Age= 41.3 ± 10.5 | Opioids within the first 28 days (Early reimbursement for an opioid prescription) | WC | Early reimbursement for an opioid prescription is associated with longer claim closure HR: 0.68 (0.53- 0.88). |
| Cifuentes 2012 [45]  USA | - Retrospective Cohort study - Average number of days between prescriptions was computed - 2 years - Generalized linear regression | Acute LBP | n=1422  F=29.6%  Age= 41.9 | Opioids  Total disability duration after the first month was used as an indicator of time off work | WC | Each additional week between opioid prescriptions predicted 14% longer disability RR: 1.14 (1.06 to 1.22).  Fewer days between opioid prescriptions were associated with shorter time off work. |
| Larson 2018 [46]  USA | - Retrospective Cohort study - Using Department of Defense healthcare utilization data between October 2012 to September 2014. - 91 - 365 days - Multivariable logistic regression | LBP (new episodes of LBP persisting > 90 days) | N=30612  F=16.5% | Opioids; Tramadol  Receipt of opioid or tramadol prescription and receipt of nonpharmacologic treatment modalities in within 30 days of LBP claim | MTF | Early opioid treatment in the first 30 days was associated with greater odds of military duty limitation OR 1.14 [1.04-1.26] |
| Lee 2016 [47]  USA | - Retrospective Cohort study - Acute LBP cases seen initially in the ED with a date of injury from 2009 to 2011 were identified within a WC dataset. - 1 year - Cox proportional hazard & Multivariate binomial log regression | Acute occupational LBP | Early Opioids  n=349  F=38.7%  No Early Opioids  n=2538  F=35.2% | Opioids  (Early opioid use was an opioid prescription originating from the initial ED visit and filled within 2 days of that visit). Duration of work disability was categorized as acute (#30 days) or chronic (>90 days). | WC | No relationship was seen between early opioid receipt and disability duration |
| Webster 2007 [48]  USA | - Retrospective Cohort study - WC claims filed between 2002 and 2003 in US states. - 2 years - Multivariate linear and logistic regression | Acute low back pain | **0 mg**  n=6651  F=28.4%  Age= 40.3±10.4  **1–140 mg**  n=437  F=28.2%  Age=39.6± 10.3  **141–225 mg**  n= 494  F=22.1%  Age=40.8± 10.7  **226–450 mg**  n =423  F=29.3%  Age= 40.6±9.5  **450+ mg**  F=4384  F=30.8%  Age= 40.7± 9.7 | Opioids  5 groups based on MEA received in the first 15 days postinjury. | WC | The average disability duration among those who received up to 140 mg MEA was not statistically different from those who received no early opioids (P = 0.61).  Those who received more than 450 mg MEA were, on average, disabled 69 days longer than those who received no early opioids (49.3 to 89.0). |
| Franklin 2008 [28]  USA | - Prospective Cohort study - Washington WC claims data and function was measured by RMDQ. - 1 year - Multivariate logistic regression | Acute back injury | N=1843  F= 592(32%)  Age=39.4 ±11.2  **No opioids**  n=1213  **Opioids 1-7 days**  n= 293  **Opioids > 7 days**  n=337 | Opioids for more than 7 days during the first 6 weeks  No opioid prescription is a reference group. | WC | Receipt of opioids for more than 7 days during the first 6 weeks were significantly associated with disability at 1 year^#^ OR: 2.2(1.5-3.1) |
| Gross 2009  [29]  Canada | - Retrospective Cohort study - Alberta WC claims from 2000 to 2005 - 1 year - Multivariable Cox regression | back sprains/ strains, dislocations, and fracture | n=137175  F=30%  Age=37 | Opioids and non-narcotics analgesia | WC | Subjects not prescribed early opioids had a higher likelihood of suspension of time loss benefits HR: 2.05 (2.00-2.10) than subjects received a narcotic prescription.  Subjects not prescribed early analgesia had a higher likelihood of suspension of time loss benefits HR: 1.50 (1.45-1.55) than subjects who received early non-narcotic analgesic medication. |

*BC: British Columbia****,*** *ED: emergency department, MEA: Morphine Equivalent Amount, MTF: Military treatment facility, NB: No benefit,* *RMDQ: Roland Morris Disability Questionnaire (It is a 24-item instrument measuring low back pain–related functional impairment: 0 indicates no functional impairment; 24 indicates maximum functional impairment.), WC: Workers’ compensation,*

*# a receipt of wage replacement benefits for temporary total disability 1 year (365 days) after claim receipt.*

**Supplementary Table 2: Prescription medicine in chronic low back pain/ Non-specified LBP and its impact on work related outcomes**

| **Study & country** | **Method**   - **Study design,** - **Data collection/ source** - **Follow-up and** - **Data analysis** | **MSD/ Injury type** | **Participants characteristics (Sample, Female, Mean age in years)** | **Prescription**  **Characteristics/exposure (group, duration)** | **Setting** | **Work outcome/summary of findings** |
| --- | --- | --- | --- | --- | --- | --- |
| Urquhart 2018  [49]  Australia | - Randomized controlled trial - Disability was assessed using the RMDQ and work absence & hindrance were assessed using the Short-Form Health and Labor Questionnaire. - 6 months - Multivariable logistic regression | Chronic Low Back Pain | Low dose Amitriptyline  n=72  F=28 (39%)  Age=53.5 ±14.2  Active comparator  n=74  F= 28 (38%)  Age=56.0 ±13.2 | **Tricyclic antidepressants**  Low dose Amitriptyline (25mg) for 6 months  Active comparator group (1 mg benztropine mesylate) for 6 months | NB | No significant difference in disability between groups at 6 months (adjusted difference, 0.98 (2.42 to 0.46)); however, there was a significant improvement in disability for the amitriptyline at 3 months (adjusted difference, 1.62 (2.88 to 0.36)).  There were insignificant differences between groups in work absence OR: 1.51 (0.43-5.38) or hindrance OR: 0.53 (0.19-1.51) at 6months. |
| Phelps 2001 [50]  USA | - Retrospective Cohort study - Patient’s charts were audited for diagnostics, therapeutics, referrals, and outcomes. - 2 years - Pearson correlation, chi-square, and ANOVA test | Low back pain | n=709  F=264(37%)  Age= 33.8±10.4 | Opioids; NSAIDs; Skeletal Muscle relaxants | WC | Several groups of medications failed to make a statistically significant impact on the probability of a successful return to work. These included: NSAIDs (p = 0.45) and muscle relaxants (p = 0.11).  No difference was observed in medicine use and return to work. |
| Chu 2012  [51]  USA | - Randomized controlled trial - Roland-Morris Disability Index at the initiation and after ^1^ month of treatment - 1-month - Mann-Whitney U test and Wilcoxon signed-rank test | Chronic non-radicular low-back pain | **Morphine**  n=69  F=25(36.2%)  Age= 44 ± 14.2  **Placebo**  n=70  F=36(51.4%)  Age= 46 ± 13.5 | Opioids  Morphine for 1 month.  The mean dose of oral morphine was 78 ± 37.5mg/day | NB | Change in Roland-Morris Disability Index after 1 month was (2.02 ± 3.06, p-value <0.001).  The morphine-treated patients experienced a 31% improvement in functional ability. |
| Mayer 2014  [52]  USA | - Prospective Cohort study - A consecutive cohort of patients outcomes were collected using a telephone interview - 1 year - Multivariate logistic regression | Chronic LBP (chronic disabling occupational lumbar spinal disorder) | **Spinal fusion surgery**  n=331  F=106(32%)  Age= 44.3 ± 8.7  **Non-fusion spinal surgery**  n=233  F=57(24.5%)  Age= 44.5 ± 9.6  **Unoperated matched comparator**  n=349  F=151(43.3%)  Age=43.5 ± 9.9 | Opioids  Opioid dependence disorder | WC | The most robust risk factor for poorer outcomes was the presence of postinjury opioid dependence disorder, which predicted lower rates of return-to-work* OR: 2.13 (1.37, 3.31) and work retention^++^ (OR: 1.94, 95% CI [1.30, 2.90]) at the one-year. |
| Preuper 2014  [53]  Netherlands | - Randomized controlled trial - Functional capacity and self-reported disability for the past 7 days were measured using functional capacity evaluation and RMDQ, respectively. - 2 weeks - Mann-Whitney U test and Wilcoxon matched-pairs signed rank sum test | non-specific CLBP | Treatment group (Acetaminophen and tramadol)  n=25  F= 18(72%)  Placebo  n=25  F= 16(64%) | Opioids/Acetaminophen | NB | Differences in functional capacity and self-reported disability between placebo and treatment group were non-significant. |
| Weil 2017  [54]  USA | - Randomized controlled trial - Work productivity and regular activities were evaluated using the Work Productivity and Activity Impairment Questionnaire Specific Health Problem (WPAI: SHP). - 12 weeks - Paired t-test & analysis of covariance | Chronic low back pain | Placebo  n=134  F=75(56%)  Age=49.3±12.24  ALO-02  n=146  F=81(55.5%)  Age=50.6±12.98 | Opioids  ALO-02, an abuse-deterrent formulation containing extended-release oxycodone and sequestered naltrexone | NB | A statistically significant difference only for mean change in percentage of activity impairment (p = 0.0040) which favored for patients treated with ALO-02 compared with those on placebo. But no difference in missed work time due to LBP. |
| Ashworth 2013  [55]  UK | - Prospective Cohort study - Patients consulting in primary care between September 2004 and April 2006 received postal RMDQ at baseline and 6 months. Opioid prescription data were obtained from electronic prescribing records. - 6 months - Multivariable linear regression and adjusted with propensity scores | LBP | **No opioid**  n=481  F= 56.1%  Age=45.77 ±9.87  **Low MED**  n=152  F=67.10%  Age=46.27 ±9.36  **Medium MED**  n=57  F=73.7%  Age=44.07± 10.69  **High MED**  n=25  F=76%  Age=47.64± 9.39 | Opioids  Baseline opioid use was defined as receipt of 1 or more prescriptions for an opioid analgesic in the 28-day period starting 14 days before the date the baseline questionnaire was sent. | NB | The mean 6-month RMDQ score(disability) was 1.18 (95% CI 0.17 to 2.19, P = 0.022) units higher for those prescribed opioids at baseline compared with those who were not prescribed any opioids. |
| Di Donato 2021  [56]  Australia | - Retrospective cohort study - Using accepted time loss claims between July 1, 2010, and June 30, 2013, in states of Victoria and South Australia. - 2.5 years - Group-based trajectory modelling and quantile regression | low back pain | No opioids  n= 6414  F= 2228 (34.7%)  Short-term  low-volume opioid group  n= 2166  F= 738 (34.1%)  Long-term  moderate-volume opioid group  n= 798  F= 285 (35.7%)  Long-term  high-volume opioid group  n= 241  F= 79 (32.8%) | Opioids  Group-based trajectory modelling to identify opioid dispensing patterns over 2.5-year period from reported LBP onset | WC | Moderate-volume and high-volume long-term dispensing groups had significantly longer wage replacement duration compared with the short-term dispensing group (median [weeks]: 126.9, 126.0, and 30.7, respectively).  High use of opioids was significantly associated with longer wage replacement duration. |
| Volinn 2009  [57]  USA | - Retrospective Cohort study - Utah WC claims of injury from January 1, 2002 to June 30, 2005 - Three and half years - Multivariable logistic regression | nonspecific low back pain | No opioid prescription  n=959  F=31%  Age= 37.0±12.3  Any schedule  n=308  F=33%  Age= 37.8±11.1  Schedule III-IV only  n=738  F=30%  Age= 36.8±11.4 | Opioids  Chronic work loss was indicated by wage compensation paid, either total or partial, for ≥ 90 days.  No opioid group is the reference group | WC | Regardless of the length of time, chronic work loss was almost twice as high for those who filled prescriptions for schedule III-IV (weak) opioids only (OR=1.9), and more than six times higher for those who filled schedule II (strong) opioids prescription (OR=6.1).  Claimants who filled opioid prescriptions over the long-term (≥ 90 days) had higher chronic work loss. The odds of chronic work loss were almost 11 times higher for those who filled prescriptions for schedule III-IV opioids only (OR=10.9) and more than 14 times higher for those who filled one or more prescriptions for schedule II opioids (OR=14.2). |
| Savych 2019  [58]  USA | - Retrospective Cohort study - Workers injured between October 1, 2008, and September 30, 2013, in the twenty-eight states. The sample includes low-back injuries with more than 7 days of lost time with prescriptions. - 24 months - Ordinary Least Square (OLS) Regression and two-stage least squares (2SLS) regression-Instrumental Variable approach | work-related low-back injuries | Any opioid prescriptions  n=836,875  Two or more opioid prescriptions  n=554,171  Three or more opioid prescriptions  n=438,223  Longer term opioid prescriptions  n=262,945 | Opioids  Data on opioid prescriptions and duration of benefits is based on payers’ records on payments made within 24 months after an injury. | WC | Implied effect % on lost time/duration of disability  Any opioid prescriptions  OLS= 50.3%  2SLS=-12.5%  Two or more opioid prescriptions (excluding longer term prescriptions) relative to no opioid prescription  OLS=76.2%, 2SLS=3.5%  Three or more opioid prescriptions (excluding longer term prescriptions) relative to no opioid prescriptions  OLS= 108.1%, 2SLS=44.8%  Longer term opioid prescriptions relative to no opioid prescriptions  OLS= 246.5%, 2SLS=158.4% |

*** RMDQ (Roland-Morris Disability Index) yields a score between 0 and 24; higher scores indicate a more pronounced disability, *Return-to-work (i.e., whether the patient returned to work at any point in the post-discharge year); ^++^work retention (whether the patient was still employed)*

**Supplementary Table 3: Preoperative opioid use and postoperative return to work in patients with spine and other surgeries in Workers’ compensation (WC) and non-WC setting**

| **Study & country** | **Method**   - **Study design,** - **Data source** - **Follow-up and** - **Data analysis** | **MSD/ Injury type** | **Surgery** | **Participants characteristics (Sample, Female, Mean age in years)** | **Prescription**  **Characteristics/exposure (group, duration)** | **Work outcome/summary of findings** |
| --- | --- | --- | --- | --- | --- | --- |
| Anderson 2015  [77]  USA | - Retrospective Cohort study - Ohio WC Claims between 1993 and 2013 - 3 years after fusion. - Multivariate logistic regression | Degenerative disk disease (DDD) | Discogenic fusion | Return to Work  n=241  F= 97 (40.2%)  Age= 43.1±8.4  Out of work  n= 796  F= 256(32.2%)  Age= 44.9±8.7 | Preoperative opioid use >1 year | OR= 0.46; (0.31-0.69) for post-operative return to work (RTW)^¶^. |
| Anderson 2016  [78]  USA | - Retrospective Cohort study - Ohio WC claims from 1993 to 2013 - 3 years after fusion - Multivariate logistic regression | Spondylolisthesis | Lumbar Fusion | Return to work  n=205  F= 56 (27.3%)  Age= 43.2±9.7  Out of work  n= 481  F= 153 (31.8%)  Age=45.1±10.5 | Preoperative opioid use >1 year | OR=0.41 (0.21-0.80) for post-operative RTW^¶^. |
| Anderson 2018  [79]  USA | - Retrospective Cohort study - Ohio WC claims from 1993 to 2013 - 3 years after fusion - Multivariate logistic regression | Degenerative disc disease | Lumbar fusion | Lumbar Discography (LD) before fusion  n= 641  F= 205 (32.0%)  Age= 43.3 ± 8.2  Control  n= 766  F= 244 (31.9%)  Age= 45.1 ± 8.8 | Preoperative opioid use >1 year | OR= 0.53 (p<0.001) for postoperative RTW^¶^. |
| Faour 2017  [81]  USA | - Retrospective Cohort study - Ohio WC claims between 1993 and 2011. - 3 Years after cervical fusion - Multivariate logistic regression | Degenerative disc disease or Radiculopathy | Multilevel Cervical Fusion | Radiculopathy  n=1068  F= 335 (33.2%)  Age= 44±8  DDD  n=441  F= 169 (38.2%)  Age= 46±8 | Preoperative opioid prescriptions | OR: 0.60 (0.48-0.75) for stable RTW^¥^ status after multilevel cervical fusion. |
| Faour 2017  [82]  USA | - Retrospective Cohort study - Ohio WC claims between 1993 and 2011. - 3 Years after cervical fusion - Multivariate logistic regression | Cervical Radiculopathy | Single-Level Cervical Fusion | STO  n=133  ITO  n=51  LTO  n=121 | Preoperative opioids based on opioid use duration,   - Short-term use (STO) (<3 months) - Intermediate-term use (ITO) (3-6 months) and - long-term use (LTO) (>6 months | Preoperative opioid use OR= 0.73 (0.55-0.98) for successful RTW*^¥^*.   - Odds of RTW were 0.49 (0.25-0.94) for ITO, and 0.40 (0.24-0.68) for LTO compared with STO group. - The odds of RTW less than 1 year after surgery were 0.43 (0.21-0.88) for ITO and 0.36 (0.21-0.62) for LTO compared with STO group. |
| Faour 2017  [80]  USA | - Retrospective Cohort study - Ohio WC claims between 1993 and 2011. - 3 Years after cervical fusion - Multivariate logistic regression | Degenerative disk disease | Single level Cervical Fusion | Opioid group  n=77  F= 29 (37.7%)  Age= 44±8.6  Control group (no previous opioid use)  n=204  F= 92 (45.1%)  Age= 44±7.6 | Preoperative Opioid use  Average length of opioid use was 474 days ±668 days. | - Patients used opioids were less likely to meet the Stable RTW OR=0.44 (0.26-0.76) and RTW within the first year after surgery (OR: 0.39; 0.22-0.70). *^¥^* - The average number of days absent from work was 255 days higher in the opioids (P=.0001). |
| Faour 2018  [83]  USA | - Case control study - Ohio WC claims between 1993 and 2011. - 3 Years after cervical fusion - Multivariate logistic regression | Cervical radiculopathy | Single-level Cervical Fusion | Opioids  n=305  F=116(38%)  Age= 40.0± 7.6  Controls (No opioids)  n=1622  F= 546 (33.7%)  Age= 41.7± 26.0 | Preoperative opioid use | - Preoperative opioid use for stable RTW status*^¥^* (OR= 0.50; 0.38-0.65) and RTW within the first year after surgery (OR=0.50; 0.37-0.66) - Stable RTW was achieved in 43.3% of the opioids and 66.6% of the control group (P = 0.05). - Permanent disability rates after surgery were higher in the opioid group (10.8%) compared with the control group (5.7%) (P < 0.05). |
| Hills 2019  [84]  USA  ** No benefit/medicaid/private insurance | - Prospective Cohort - Linked data of opioids and registry for elective spine surgery between 2010 and 2017. - 9-months for preoperative opioids & 1-year for Postoperative outcomes - Multivariable logistic regression | Back pain (cervical and lumbar) | Spine surgery | No Preop Chronic  Opioid Therapy  n=1683  F= 858 (51%)  Preop Chronic  Opioid Therapy  n= 445  F= 240 (54%) | Preoperative opioid use   - Chronic opioid therapy is defined using the common duration of 3 months. | - Preoperative Chronic Opioid Therapy OR= 1.34 (0.8 - 2.25) and High-Preoperative Opioid Dosage 1.11 (0.6 - 2.08) was not associated with the outcome "Not returned to work status". - Patients treated with chronic opioids prior to elective spine surgery are less likely to achieve meaningful improvements at 1-year in function. But not associated with return-to-work status |
| Kho 2017  [89]  USA  ***  Not spine surgery | - Cross sectional study - Medical records of all WC patients who underwent open CTR surgery over a 5-year period. - Analysis: T-test and ANOVA | Carpal tunnel syndrome | Carpal tunnel release (CTR) surgery | N=125  F=75(60%)  **Preoperative opioid use**  n=12  **no preoperative opioid use**  n=113 | Preoperative opioids | *** Return to work in preoperative opioid use is 21.7 weeks ±15.7 vs 11.6weeks ±10.4 in no preoperative opioid use, p=0.05 |
| O'Donnell 2018  [86]  USA | - Retrospective Cohort study - Ohio WC claims for injury between 2005 and 2012 - Follow-up: 3 years - Multivariate logistic regression | Lumbar disk herniation (LDH) | Lumbar Diskectomy (single level) | **No opioid**  n= 566  F= 114 (20.1%)  Age=40.9 ± 10.2  **STO**  n= 126  F= 29 (23.0%)  Age= 40.0 ±10.9  **MTO**  n= 315  F= 92 (29.2%)  Age= 39.4 ± 9.7  **LTO**  n= 279  F= 74(26.5%)  Age= 39.1± 9.3 | Preoperative opioid use  Short term preoperative opioid use (STO)  < 14 days of preoperative opioid use.  Moderate preoperative (MTO) opioid uses 14- 90 days of opioid use  LTO was defined using >90 days of e opioid use. | - RTW*^¶^* was significantly lower for the LTO group (36.9%) than the no opioid (64.1%; P < 0.01), the STO (63.5%; P < 0.01), and the MTO (52.7%, P < 0.01) populations. - Preoperative Opioid use was an independent negative predictor of RTW with OR= 0.54 (0.39-0.75). |
| Tye 2017  [87]  USA | - Retrospective Cohort study - Ohio WC claims 1993 and 2013. - At least 3 years - A multivariate regression | Degenerative Lumbar Stenosis | Lumbar decompression | **Short-Term Opioid Cohort**  n=60  F=22 (37%)  Age= 52.1±8.8  **Long-Term Opioid Cohort**  n=80  F= 19 (24%)  Age= 47.2 ±10.7 | Preoperative Opioid use   - short-term use (<3 months opioid use duration) and - long-term use (>3mnts opioid use duration) prior to surgery. | - Patients with short term opioid use had a significantly higher RTW*^¶^* rate compared with long term opioids [25/60 (42%) vs. 18/80 (23%); P=0.01]. - Long term opioid use was negative predictor of RTW OR: 0.35, (0.13-0.89). |
| Zakaria 2020  [88]  USA  **no benefit scheme | - Prospective Cohort study: - Using Spine registry data and Oswestry Disability Index (ODI) - 2 years - Multivariate generalized estimating equation models | Back pain (spondylosis and intervertebral disc disorders) | Lumbar Fusion | **Less than 6 weeks**  n=735  F=412(56%)  Age= 62.4 ± 12.5  **6 wk-3 months**  n=421  F=236(56%)  Age= 61.0 ± 12.7  **3-6 months**  n=562  F=330(59%)  Age= 59.7 ± 12.8  **Greater**  **than 6 months**  n=3314  F=1869(56%)  Age= 58.8 ± 12.4 | Preoperative Opioids   - Opioid-naive (no usage), - New users (<6 weeks), - Short-term users (6 weeks-3 months), - Intermediate-term users (3-6 months) - Chronic users (>6 months). | - New opioid users to show improvement in ODI at 90 days RR: 1.25 (1.14 to 1.38), 1 year RR: 1.15 (1.07 to 1.23), and 2 years RR: 1.22 (1.10 to 1.37). - Short-term opioid users to ODI improvement at 90 d (RR: 1.15 (1.06 to 1.24). - Chronic opioid users to ODI improvement at 90 days RR: 0.89 (0.84 to 0.95), 1 yr. RR: 0.87 (0.80, 0.95) and 2 yrs. RR: 0.82 (0.71 to 0.94). - Chronic opioid users were less likely to return to work at 90 d RR: 0.83 (0.76 to 0.91). |
| McMillan 2022  [85]  Australia | - Retrospective cohort study. - Victorian WC from 2008 to 2016 - 24 months after LSF surgery - Multivariate multinomial logistic regression | Non-catastrophic workplace injury | Elective Lumbar Spinal Fusion (LSF) | N=874  F=272(31.1%)  Age=45.6±9.6 | Preoperative opioid use  Morphine Equivalent Daily Dose (MEDD) in the 3-month period,   - MEDD of 0 mg. - ≤40 mg/day (low dose) - > 40 mg/day (high dose) | Opioid use prior to surgery is associated with having only partial or no work capacity at 24 months (high dose: OR (3.08: 1.50–6.32)) |

*¶ A RTW was defined as employment within 2 years of fusion/surgery, with subsequent sustained work greater than 6 months in the following year*

*¥Stable RTW is if they returned to work and maintained continuous at-work status for at least 6 months within 3-year period after surgery.*

**** RTW duration was based on the date the patient was cleared to RTW by the treating surgeon*

***Supplementary Table 4: Peri- and post-operative/procedure prescription medicine use and its impact on work related outcomes in patients with musculoskeletal conditions***

| **Study & country** | **Method**   - **Study design,** - **Data collection/ source** - **Follow-up and** - **Data analysis** | **MSD/ Injury type** | **Surgery /procedure** | **Participants characteristics (sample, Female, Mean age in years)** | **Prescription**  **Characteristics/exposure (group, duration)** | **Setting** | **Work outcome/summary of findings** |
| --- | --- | --- | --- | --- | --- | --- | --- |
| Anderson 2015  [90]  USA | - Retrospective Cohort study - Ohio WC Claims between 1993 and 2013 - 3 years after fusion - Multivariate logistic regression | Degenerative Disc Disease and discogenic LBP | Lumbar fusion | **COT Group**  n=575  F= 192 (33.4%)  Age = 44.5 ± 8.4  **TOT Group**  n=427  F= 148 (34.7%)  Age= 44.4 ± 8.9 | **Postoperative Opioid use**  Chronic opioid Therapy (COT): Opioid analgesics for greater than 1 year after the 6-week acute period after fusion.  Temporary Opioid therapy (TOT): opioid supplied for less than 1 year following a 6-week postoperative period | WC | Return to work was negatively associated with COT (OR of 0.38 (0.25-0.57). |
| Burke 2010  [91]  Ireland | - Randomized controlled trial - Patients completed RMDQ and Medical Outcomes Study Short Form 36 (SF-36) before and 3 months after surgery - 3 Months - t-test and ANOVA | Radicular low back pain (3-month to 12-month duration) | Lumbar discectomy | Placebo  n=20  F= 9(45%)  Age= 41±12.4  Pregabalin  n=18  F= 5(27.8  Age= 37±7.8 | **Pregabalin perioperative** (300 mg at 90 minutes preoperatively and 150 mg at 12 and 24 hours postoperatively) | NB | The RMDQ score was less at 3 months postoperatively in pregabalin group (2.7 ±2.4) than the placebo group (5.6 ± 4.8).  All the patients in the pregabalin group were returned to work at 3 months, compared with 75% of the placebo group. |
| Khurana 2014  [92]  India | - Randomized controlled trial - Postoperative functional outcome recorded using questionnaires of Prolo score, Oswestry Disability Index score from preoperative period to 3 months postoperatively - 3 months - Kruskal-Wallis test | Chronic LBP | Lumbar discectomy | **Gabapentin**  n=30  F=8(26.67%)  Age= 49 ± 10.4  **Pregabalin**  n=30  F=6(20%)  Age= 46.9 ± 10.1  **Placebo**  n=30  F= 8(26.67%)  Age= 47.1 ± 10.7 | **Gabapentin and Pregabalin Perioperative use**   - Group A received 300 mg of gabapentin, - group B received 75 mg of pregabalin, - group C received a placebo 1 dose 1 hour before surgery   **+**  and 8 hourly for 7 days | NB | Pregabalin is associated with improved functional outcomes 3 months after lumbar discectomy followed by gabapentin |
| Rudbeck 2013  [94]  Denmark | - Retrospective Cohort study - 3 state registries on employment/benefits, medication, and industrial injuries - 5 years - Multivariate logistic regression | Shoulder impingement syndrome | Arthroscopic subacromial decompression (ASD) | n=615  F=329 (53.5%)  Age=46.9 | Perioperative use  Opioids; NSAIDs; Antidepressant; Acetaminophen/Paracetamol  Preoperative for the 6 months before and Pos- operative 1 year after ASD | NB; WC; SAS, UB | OR of sick leave benefits 1 year after ASD for painkillers 2.18 (1.37-3.46), strong painkillers/ opioids 3.78 (2.32-6.16)  At 5 years after ASD, 35 (22.6%) were receiving permanent benefits. Use of opioids at any time during a 1-year period after ASD (24.80 [7.05-87.18]) were a predictor. |
| Kraus 2021  [95]  USA | - Prospective Cohort study - Registry from 2012 to 2016 and administered a preoperative and 4 postoperative surveys - 2-year - A linear mixed-effects model | Rotator cuff injury | Arthroscopic Rotator Cuff Repair | **Ibuprofen group**  n=182  F=89(49%)  Age= 55.93±9.72  **Opioid group**  n=281  F=117(42%)  Age= 57.01± 9.13 | Post-operative Opioids; NSAIDs | NB & WC | There were no significant differences in all patient-reported functional outcomes between the group prescribed ibuprofen and opioids at 1 and 2 years after surgery |
| Webster 2004  [93]  USA | - Case series - WC records between December 1, 1998, and February 29, 2000 - 24 months - Multivariate logistic regression | Low back pain | Lumbar intradiscal electrothermal therapy (IDET) procedure | n=142  F=47 (33.1%) | Opioids  Pre-procedure; Post procedure | WC | Not returning to work was associated with using narcotics 3 months before IDET OR=0.20 (0.07-0.57). |

*WC: Workers’ compensation, NB: no benefit scheme, SAS: sickness absence scheme, UB: unemployment benefit, RMDQ:* Roland Morris Disability Questionnaire

**Subjects made a RTW within 2 year of fusion and sustained it for greater than 6 months out of the following year*

***Supplementary Table 5: Prescription medicine use in multiple body part musculoskeletal conditions (Osteoarthritis,* limb, shoulder, non-specified and multiple body part injuries*) and its impact on work related outcomes***

| **Study & country** | **Method**   - **Study design,** - **Data collection/ source** - **Follow-up and** - **Data analysis** | **MSD/ Injury type** | **Participants characteristics (Sample, Female, Mean age in years)** | **Prescription**  **Characteristics/exposure (group, duration)** | **Setting** | **Work outcome/summary of findings** |
| --- | --- | --- | --- | --- | --- | --- |
| Goorman 2000  [59]  USA | - Case series - SF-36 Health Survey completed before and 6 months after treatment. - 6 months - t-test | Knee Osteoarthritis | n=61  F=35(57.4%)  Age=65.8±11.65 | Intra-articular Hylan G-F 20  Patients received 3 weekly 2cc Intra-articular injection | NB | Statistically significant improvement (p <0.001) in Physical Functioning, Role-Physical, Bodily Pain, Social Functioning, and Role-Emotional categories on a 6-month follow-up survey. |
| Tillander 2018  [36]  Marathon runners from  Multiple countries | - Cross sectional study - A web-based questionnaire of runners registered for a marathon. The 1-year prevalence of time-loss injury and illness was assessed - Chi squared test | Knee, foot, Achilles tendon, hip, thigh, calf, spine, groin, ankle, toes and lower leg injuries | n=161  F= 26(26%) | NSAIDs  Time loss injury as runners reporting avoidance of running for more than 3 weeks because of injury in the past 12 months. | NB | Runners who reported NSAID use in the past 12 months reported fewer time-loss injuries than non-users (p=0.003). |
| Sundstrup 2017  [61]  Denmark | - Prospective Cohort study - Employed wage earners that were not on long-term sick leave during 2009 and 2010. Long-term sickness absence (LTSA) defined as having registered for at least 6 consecutive weeks. - 2-year - Multivariate Cox-regression | Musculoskeletal pain in the low back, neck/shoulder, and hand/wrist | n=9544  F= 5065 (53.1%)  Age= 43.2±11.8 | Use of pain medication for periods of at least 14 days within the last year was used as regular use of medicine. | SAC | Regular use of over-the-counter HR: 1.44 (1.13-1.83) and doctor-prescribed HR: 2.18 (1.67-2.86) pain medication were prospectively associated with LTSA.  Regular use of doctor-prescribed pain medication due to musculoskeletal pain was a stronger predictor for long-term sickness absence compared with the use of over-the-counter pain medication |
| Johnston 2016  [62]  USA | - Retrospective Cohort study - US insurance claims linked administrative data on workers compensation and short-term disability between 2004 and 2012. - 1 year - Multivariable generalized linear models | Fracture (skull, spine, upper& lower limb), dislocations, sprains, strains, open wounds, and burns. | STD (Opioid Abuse)  n= 386  F=23.6%  Age= 42.1 ± 9.6  STD (No Opioid Abuse)  n=71622  F=37.8%  Age= 44.8 ± 10.2  WC (Opioid Abuse)  n=189  F=21.2%  Age= 43.6 ±9.3  WC (No Opioid Abuse)  n=35778  F=33.4%  Age= 45.5 ±9.6 | Opioids; Benzodiazepines; NSAIDs; Skeletal Muscle relaxants; Tricyclic antidepressants; Selective serotonin reuptake inhibitors; Steroids; Acetaminophen | WC | Slightly attenuated differences in mean days lost work in the STD sample between diagnosed opioid abuse and no opioid abuse (adjusted mean days lost from work 76 vs. 60; adjusted mean lost wages $9718 vs. $7661). |
| Thakkar 2021  [60]  USA | - Retrospective Cohort study - US Employers' healthcare/ disability insurance claims from January 1, 2014, to December 31, 2017 - Multivariable regression | Osteoarthritis (OA) | OA Cohort  n= 144355  Control Cohort  n= 392639 | Opioids | WC | Employees prescribed opioids had significantly higher estimated lost workdays and payments. For lost workdays, the overall estimated IRR for opioids was about 2.4 for WC and 5.5 for STD. |
| Szucs 2022  [63]  Australia | - Retrospective Cohort study - Victorian WC Claims lodged between 2008-2018. Duration of time loss was defined using number of compensated days. - maximum of 130 weeks - Binary and multinomial logistic regression, Cox regression | Lower limb Injury | N= 51334  F= 16,582 (32.3%)  Dispensed at least  one opioid  n= 12017  F=3617 (21.8%) | Opioids | WC | All types of opioids were associated with increased duration of time loss. The strength of opioid increased the median compensated days increased.  A combination of strong and weak opioids was related to the longest duration of time loss with HR=0.28 (0.27, 0.29), followed by strong opioids alone HR=0.46 (0.45, 0.48) and weak opioids alone HR=0.57 (0.55, 0.59) as compared with no opioids. |
| Berecki-Gisolf 2013  [64]  Australia | - Retrospective cohort study - Persons injured in motor vehicle accidents from 2005-to 2007 and - 17 months - Logistic regression | Dislocations, fractures, sprains/strains, and whiplash injury | n=5970  F=1847 (37%) | Opioids within 10 days of a traffic accident | WC/ TAC | Receiving at least one prescription was associated with receiving benefits after 6 months OR: 2.1(1.5-2.9).  Receiving at least one prescription was associated with receiving loss of earning capacity payments^$^ OR: 2.2 (1.5-3.1). |
| Haight 2020  [65]  USA | - Retrospective Cohort study - WC claims of Washington Department of Labor and Industries - 5 years - Multivariable logistic regression | Multiple types (Fracture, Strain/sprain/ tear, other traumatic injuries) on the neck, back, upper and lower extremity | n=83150  F= 25,857 (31.1%)  Age=36.9 | Prescribed at least One Opioid within the First 6 Weeks of Injury  Composite high-risk opioid prescribing indicator | WC; SS and DP | Exposure to high-risk opioid prescribing was associated with 3.12 times higher odds (2.97, 3.27) of more than 90 days of time loss, 2.88 times higher odds (2.71, 3.06) of more than 1 year of time loss, 3.11 times higher odds (2.39, 4.05) of total permanent disability, and 2.76 times higher odds (2.26, 3.38) of Social Security offset (SSO) when compared with low-risk opioid prescribing exposure |
| Kidner 2009  [66]  USA | - Prospective Cohort study - Patients admitted to interdisciplinary functional restoration program. ODI and SF-36 tools were used to measure function at beginning and end of the study. - 1 Year - Linear and logistic regression | Chronic Disabling Occupational Musculoskeletal Disorders (least one extremity bone/joint or spinal region injury). | Opioid use  n=596  F=48.2%  Age=44.1 ± 9.3  No Opioid use  n=630  F=50%  Age= 43.4+1 | Opioids | WC; SS; SSI | High opioid use was significantly related to lower rates of return to work and work retention (p < 0.05). Thus, the Very High subgroup was 11.6 times as likely as the No subgroup to be receiving Social Security Disability Income/ Supplemental Security Income benefits at the time of the one-year follow-up OR: 11.62 (3.51 to 38.46) |
| MacLaren 2006  [67]  USA | - Prospective Cohort study - Patients completed a multidisciplinary rehabilitation program Return-to-work outcomes were obtained via phone calls 6 months posttreatment - 6 months - Repeated ANOVA and chi-square test | Low back pain, neck and shoulder pain, upper and lower extremity pain | Patients Taking  Opioids  n=70  F=45.71%  Age= 41.61±7.79  Patients not Taking  Opioids  n=57  F=49.12%  Age= 39.47±8.39 | Opioids | WC | No significant differences between opioid and nonopioid users on physical, or return-to-work outcomes |
| White 2020  [68]  USA | - Retrospective Cohort study - Using OptumHealth Care Solutions, Inc. claims from January 1, 2012, to March 30, 2016 - 12 months - Multivariate logistic regression /propensity score matching | Majorly MSK population (Trauma, arthritis, Back and neck pain, Fibromyalgia) | Matched cohort of OUD  N=2311  F=1009(43.6%)  Age=36.4 ±15.1  Matched cohort of no OUD  N= 2311  F=1050(45.4%)  Age=50.6±12.98 | Opioids  OUD (opioid use disorder | PI/  SI | Mean (SD) total work-loss days (days of absence related to medical visits and disability/waiting for disability) were significantly higher for employees with OUD compared with controls (27.5 [31.1] vs 15.7 [20.0]; P < 0.001], translating into mean (SD) annual total work loss costs of $8193 ($14,694) and $5438 ($13,683) (P < 0.001), respectively. |
| Hunt 2019  [26]  USA | - Retrospective Cohort study - WC claims data with injury dates between 2008 and 2013 with follow-up to Dec 31, 2017. - At least 4 years - A multivariable logistic regression | Chronic pain | 22,383 work-related indemnity claims | Opioids; Benzodiazepines; Antidepressants | WC | The OR for Claims with anti-depressant, opioid, or benzodiazepine prescriptions were 2.24 (2.00 to 2.51), 1.14 (1.02 to 1.27), and 1.38 (1.23 to 1.54) times more likely to remain open at the end of the study. |
| Lavin 2014  [17]  USA | - Retrospective Cohort study - Using Louisiana WC lost time claims from 1999 to 2002. - 7 Years - Multivariate logistic regression | Work related injuries | N=11,394  F=27.5%  Age=40.1 | Opioids; Benzodiazepines | WC | The OR for benzodiazepines being associated with claim costs (indemnity claim for lost time) >100,000$ was 2.74 (2.31 to 3.26) times higher than claims without benzodiazepines. However, benzodiazepines do not contribute to these claim costs as much as short acting opioids OR = 4.69 (3.80-5.77) and long-acting opioids OR = 14.24 (10.81-18.76). |
| Tao 2015  [69]  USA | - Retrospective Cohort study - Linked data of prescription and Louisiana WC lost time claims between 1999 to 2002 and claims were closed before December 31, 2009. - Multivariate logistic regression | Non-specified work-related injury | N=11,190  Never used opioids  n=6309 (56.4%)    Prescribed opioids or the psychotropic drugs  n=4881 (43.6%) | Opioids;  Psychotropic: Antidepressant; Anti-anxiety agent (anxiolytics, minor tranquilizers), antipsychotic (neuroleptic, major tranquilizer), and hypnotics (sedative hypnotic) | WC | OR of claim costs ≥$100,000 compared with claimants who were never prescribed opioids were 4.3 for short-acting opioids only (3.49 to 5.33); 8.6 for any use of long-acting opioids (6.32 to 11.61); 2.8 for any use of hypnotics (2.29 to 3.49); 2.6 for any use of antipsychotics (1.45 to 4.60); 1.6 for any use of anti-anxiety agents (1.29 to 1.92); and 2.9 for any use of antidepressants (2.43 to 3.53). |
| Tao 2015  [70]  USA | - Retrospective Cohort study - Linked data of prescription and Louisiana WC lost time claims between 1999 to 2002 and claims were closed before December 31, 2009. - Multivariate Logistic regression and Cox Proportional Hazard Model | Non-specified work-related injury | N=11394 | Opioids; Antidepressants; hypnotics, antidepressants, or antianxiety agent | WC | The ORs for claim duration at least 3 years was 1.36 (1.21-1.53) for claimants prescribed SA opioids, 1.71 (1.33-2.19) for HAA (hypnotics, antidepressants, or antianxiety agents), and 2.30 (1.19-4.47) for LA opioids during the same time.  The initial 60 days of prescriptions for psychotropic medications were significantly associated with a final claim cost at least $100,000. Odds ratios were 1.88 for short-acting opioids, 2.14 for hypnotics, antianxiety agents, or antidepressants, and 3.91 for long-acting opioids, respectively. |
| Nkyekyer 2018  [71]  USA | - Cross-sectional study - Washington State WC claims from 2012-2015 linked with prescription monitoring data of 12 months before injury and 90 days after injury - Chi-squared test | Acute occupational injuries such as strains/ sprains, fractures, burns, abrasion | N=313,543 claims  F= 32.9%  Age= 38.5±12.6 | Opioids; Benzodiazepines | WC | Compensable claims were higher among cases with any pre-injury opioid use compared to cases without opioid use before injury (28.6% vs. 19.5%, p < 0.0001). compensable claims were also higher among cases with any benzodiazepine use before injury compared to cases without (29.7% vs. 20.0%, p< 0.0001) |
| Brede 2012  [72]  USA | - Prospective Cohort study - Using patients referred to rehabilitation center - 1 year after discharge - Multivariate logistic regression | CDOMD | Work retention (WR) group  n= 1486  F= 45.7%  Age= 45.9 ± 9.4  Non-WR group  n=364  F= 54.1%  Age= 49.3 ± 8.7 | Opioids | NB; SC & Supplemental income | Dependence on opiate pain medications was associated with failure to retain work^@^ after 1 year (OR=1.43; 95% CI, 1.02-2.00) |
| Franklin 2019  [73]  USA | - Longitudinal ecological study - Washington state Department of Labor and Industries Data (2013-2017) - Quarterly (3 months) - Descriptive statistics | Chronic pain following injury |  | Opioid prescriptions between 6- and 12-weeks following injury, an indicator of persistent opioid use | WC | A sustained drop in persistent opioid use (following the implementation of limiting opioid use policy-prior authorization to use opioids beyond six weeks) from nearly 5% in 2013 to less than 1% in 2017. This reduction was associated with the reversal of the increased lost work time patterns seen in previous years. |
| Lavin 2016  [74]  USA | - Retrospective Cohort study - Using Louisiana WC lost time claims from 1999 to 2002. Claimants with a claim duration of >180 days were selected. - At least 3 years claim duration - Multivariate logistic regression | Non-specified work-related compensable injury | N=7211 | Opioids  Prescriptions for the opioids were observed in four-time windows: 0 to 30 days, 31 to 90 days, 91 to 180 days, and 181 to 360 days. | WC | OR for claim cost at least $100,000 and duration at least 3 years were not statistically different between groups prescribed opioids less than 30 days and those not prescribed opioids. Claims with short-acting opioids continued after 180 days; the odds ratios for claims cost of at least $100,000 and duration at least 3 years were 6.21 (5.30 to 7.28) and 3.32 (2.94 to 3.74), respectively. |
| Lavin 2017  [75]  USA | - Retrospective Cohort study - Using Johns Hopkins WC claims of injured workers. Opioid supply days minus temporary total days (TTDs) are a surrogate for the number of days at work while taking opioid medication - Descriptive statistics | Occupational injury | N=4994  F=70.9%  Age=53.1 | Opioids  1) at least 3 consecutive months prescribed (chronic opioid therapy; COT);  2) less than 3 consecutive months prescribed (acute opioid therapy; AOT); and  3) no opioids prescribed | WC | The average COT claimant had been prescribed opioid medication for 808.2 days after being released for work. In contrast, the average AOT claimant was released to work after opioid prescribing had been discontinued because the average TTD (182.3 days) was 153.8 days longer than the average number of days those opioids were prescribed (28.5 days). |
| Dersh 2008  [76]  USA | - Prospective cohort study - Patients attending functional restoration program including detoxification from opioids. Interview administered 1-year post discharge - 1 year - Multivariate regression analysis | Disabling occupational spinal disorders | Non-ODD  n= 1124  F= 432(38.4%)  Age= 41.87±9.80  Non-ODD  n=199  F= 75(37.7%)  Age= 41.97±8.44 | Opioid dependence | WC | ODD patients were approximately 2 times more likely than non-ODD patients to fail to return to work OR=1.7(1.0, 2.7, P=0.043) and retain work after 1 year OR= 2.0(1.3-3.0, P= 0.002) |

*CDOMD-Chronic disabling occupational musculoskeletal disorder, DP-Disability Pension, IRR-incident rate ratios, NB: no benefit scheme, ODD-Opioid dependence disorder, ODI- Oswestry Disability Index, PI/SI-privately insurance/self-insurance beneficiaries, SA-Short acting, SAC-Sickness Absence scheme, SF-36 -Short Form-36 Health Survey, SS: social security, STD-short term disability, TAC- Transport Accident Commission, WC-workers’ compensation*

*$- Return to work was defined as the final cessation of ‘loss of earnings’ payments within the follow-up time of 17 months, ^@^Work retention (a patient's ability to both obtain and retain employment) at 1 year after discharge from treatment*

**Search strategy**

A MEDLINE, EMBASE and PsycINFO database search strategy via Ovid platform

**Supplementary Table 6: Full search strategy employed via Ovid platform**

| **PCC** | **Search Guide** | **#** | **Search term** |
| --- | --- | --- | --- |
| **Population/**  **Problem** | Workers,  Injury, MSDs |  | Worker* |
|  |  |  | Injured adj3 worker* |
|  |  |  | Compensation adj3 claimant* |
|  |  |  | Wounds and Injury |
|  |  |  | Injur* |
|  |  |  | Work related adj injur* |
|  |  |  | Musculoskeletal adj injur* |
|  |  |  | Musculoskeletal adj disorder |
|  |  |  | Musculoskeletal diseases |
|  |  |  | Occupational injury |
|  |  |  | Low back pain |
|  |  |  | Back pain |
|  |  |  | Back adj3 pain |
|  |  |  | Arthritis |
|  |  |  | Sprains and strains |
|  |  |  | Tendonitis |
|  |  |  | Herniated disks |
|  |  |  | Sciatica |
|  |  |  | Fracture |
|  |  |  | Dislocation |
|  |  |  | Osteoarthritis |
|  |  |  | Contusions |
|  |  |  | Abrasions |
|  |  |  | Tension Neck Syndrome |
|  |  |  | Thoracic Outlet Compression |
|  |  |  | Rotator Cuff injuries OR shoulder pain |
|  |  |  | Epicondylitis |
|  |  |  | Radial Tunnel Syndrome |
|  |  |  | Digital Neuritis |
|  |  |  | Trigger Finger / Thumb |
|  |  |  | Degenerative adj Disc adj Disease |
|  |  |  | Ruptured Disc |
|  |  |  | **1 OR 2… OR 32** |
| **Concept (Prescription medicine use and work-related outcomes and/or Safety outcomes)** | Prescription medicine |  | Medicine |
|  |  |  | Medication |
|  |  |  | Drug |
|  |  |  | Medic* adj3 use |
|  |  |  | Prescription adj medicine |
|  |  |  | Prescription drug* |
|  |  |  | Opioid* |
|  |  |  | Opiate* |
|  |  |  | Codeine |
|  |  |  | Hydrocodone |
|  |  |  | Oxycodone |
|  |  |  | Meperidine |
|  |  |  | Tramadol |
|  |  |  | dihydromorphinone |
|  |  |  | Hydromorphone |
|  |  |  | Morphine |
|  |  |  | Oxymorphone |
|  |  |  | Fentanyl |
|  |  |  | Methadone |
|  |  |  | Pethidine |
|  |  |  | Antidepressant* |
|  |  |  | TCA or tricyclic antidepressants |
|  |  |  | Amitriptyline |
|  |  |  | Nortriptyline |
|  |  |  | Protriptyline |
|  |  |  | Maprotyline |
|  |  |  | Imipramine |
|  |  |  | Desipramine |
|  |  |  | Amoxapine |
|  |  |  | Trimipramine |
|  |  |  | Doxepin |
|  |  |  | SSRI or selective serotonin reuptake inhibitor |
|  |  |  | Fluoxetine |
|  |  |  | Citalopram |
|  |  |  | Escitalopram |
|  |  |  | Sertraline |
|  |  |  | Paroxetine |
|  |  |  | Fluvoxamine |
|  |  |  | Benzodiazepine |
|  |  |  | Diazepam |
|  |  |  | Bromazepam |
|  |  |  | Clonazepam |
|  |  |  | Alprazolam |
|  |  |  | Lorazepam |
|  |  |  | Oxazepam |
|  |  |  | Prazepam |
|  |  |  | Triazolam |
|  |  |  | Estazolam |
|  |  |  | Flurazepam |
|  |  |  | Analgesi* |
|  |  |  | NSAIDs or non-steroidal anti-inflammatory drugs |
|  |  |  | Naproxen |
|  |  |  | Diclofenac |
|  |  |  | Etodolac |
|  |  |  | Sulindac |
|  |  |  | Ketorolac |
|  |  |  | Celecoxib |
|  |  |  | Etoricoxib |
|  |  |  | Parecoxib |
|  |  |  | Ibuprofen |
|  |  |  | Ketoprofen |
|  |  |  | Flurbiprofen |
|  |  |  | Oxaprozin |
|  |  |  | Indomethacin |
|  |  |  | Aspirin OR ASA |
|  |  |  | Meloxicam |
|  |  |  | Piroxicam |
|  |  |  | Mefenamic acid |
|  |  |  | Nabumetone |
|  |  |  | Skeletal Muscle relaxants |
|  |  |  | Cyclobenzaprine |
|  |  |  | Methocarbamol |
|  |  |  | Acetaminophen or Paracetamol |
|  |  |  | Narcotic* |
|  |  |  | Psychotropic* |
|  |  |  | Anticonvuls* |
|  |  |  | **34 OR 35 OR---OR 109** |
|  | Work-related outcomes |  | Return to work |
|  |  |  | Time off work |
|  |  |  | Absenteeism |
|  |  |  | Time loss |
|  |  |  | Work incapacity |
|  |  |  | Work disability |
|  |  |  | Work impairment |
|  |  |  | Work status |
|  |  |  | Employment status |
|  |  |  | Work capacity |
|  |  |  | Work function |
|  |  |  | Presenteeism |
|  |  |  | Functional status |
|  |  |  | Functional limitation |
|  |  |  | Functional impair* |
|  |  |  | Functional capacity |
|  |  |  | Functional ability |
|  |  |  | Physical functioning |
|  |  |  | Work performance |
|  |  |  | Work productivity |
|  |  |  | Work effective* |
|  |  |  | Missed workday* |
|  |  |  | Disability leave |
|  |  |  | Sick* leave |
|  |  |  | Medical leave |
|  |  |  | Duration of unemployment |
|  |  |  | Wage replacement duration |
|  |  |  | Time to return to work |
|  |  |  | Delayed return to work |
|  |  |  | Lost work time |
|  |  |  | Productiv* loss |
|  |  |  | **111 OR 112 OR---OR 141** |
| **Context** | Benefit scheme setting |  | Compensation adj3 claim* |
|  |  |  | Worker* adj3 compensation adj claim* |
|  |  |  | Employer adj2 entitl* |
|  |  |  | Ill* benefit |
|  |  |  | Reimburse* adj medicine |
|  |  |  | Disability support |
|  |  |  | Disability claim* |
|  |  |  | Disability compensation |
|  |  |  | Disability payment* |
|  |  |  | Disability insurance |
|  |  |  | Disability benefit |
|  |  |  | Wage replacement |
|  |  |  | Sick* benefit* |
|  |  |  | Sickness absence benefit |
|  |  |  | **143 OR 144 OR…156** |
| **Final search**  **results** |  |  | **33 AND 110 AND 142 AND 157** |
